# Optimally adjusted last cluster for prediction based on balancing the bias and variance by bootstrapping

**DOI:** 10.1371/journal.pone.0223529

**Published:** 2019-11-04

**Authors:** Jeongwoo Kim

**Affiliations:** 1 Korea Maritime Institute, Busan, Republic of Korea; 2 Biomedical Research Center, Asan Institute for Life Sciences, Seoul, Republic of Korea; Rutgers University, UNITED STATES

## Abstract

Estimating a predictive model from a dataset is best initiated with an unbiased estimator. However, since the unbiased estimator is unknown in general, the problem of the bias-variance tradeoff is raised. Aside from searching for an unbiased estimator, the convenient approach to the problem of the bias-variance tradeoff may be to use the clustering method. Within a cluster whose size is smaller than the whole sample, we would expect the simple form of the estimator for prediction to avoid the overfitting problem. In this paper, we propose a new method to find the optimal cluster for prediction. Based on the previous literature, this cluster is considered to exist somewhere between the whole dataset and the typical cluster determined by partitioning data. To obtain a reliable cluster size, we use the bootstrap method in this paper. Additionally, through experiments with simulated and real-world data, we show that the prediction error can be reduced by applying this new method. We believe that our proposed method will be useful in many applications using a clustering algorithm for a stable prediction performance.

## Introduction

When analyzing relationships among variables, including random variables with an equation of **y** = *f*(**x**)+**e**, where **e** is a random vector of a certain distribution with zero mean and preset covariance, two main problems are generally encountered: one is related to *f*(**x**) and the other to **e.** The latter mainly includes heteroscedasticity and serial correlation problems, which induce efficiency of the estimator to decrease, thereby hindering rigorous hypothesis testing. The former, which is related to the misspecification problem leading to biased estimator, frequently presents a critical problem. In numerous applications, unless an underlying theoretical foundation for an approximation model has been rigorously established or is widely acknowledged in a related area, one is prone to obtaining a biased estimator.

If the purpose of a study is to verify an exact relationship among the given variables of a certain dataset, the first priority is to set the approximation model with the best fit—i.e., an unbiased model specific to the given dataset. However, for prediction purposes, one may not struggle to obtain an unbiased estimator to yield the smallest error metric by considering only the given dataset. In fact, focusing only on the given dataset may result in the overfitting problem, which hinders accurate prediction; therefore, a biased approximation model can sometimes be useful for the purpose of prediction.

Using a biased approximation model, the more instances we use, the greater the predictor bias we obtain. Therefore, with a biased approximation model, a subsampling approach could yield a more accurate performance up to the point where the gain from the reduced bias exceeds the loss from the increased variance [1]. Conversely, the subsampling approach could have serious drawbacks when the increased variance considerably exceeds the reduced bias because the convergence rate of variance is generally *O*(*N*^−1^), where *N* is the sample size. Moreover, searching for every neighbor reference of a target point, such as the k-nearest neighbor and moving average methods, is computationally expensive [2]. Considering these factors, a more efficient method would be to partition the data into clusters. Clustering methods are easy to implement and extensively used as one of the unsupervised learning methods. However, in general, the number of clusters is not analytically obtained, but numerically searched in a set of candidate numbers. Therefore, it is prone to lead to the overfitting problem [3, 4]. With the effect of the overfitting problem, a poor cluster would cause high variance [5] because the small size of the subsample usually displays a high variance [6, 7]. Thus, determining how to adequately adjust the size of the cluster would be a key approach to overcome the high variance and the overfitting problem. In particular, to adjust the size of the cluster for prediction, we may consider the cluster close to a prediction target, unlike the usual clustering method fitting a whole dataset. In the current study, the term *last cluster* indicates the cluster located closest to a prediction target after partitioning the data into clusters using a method such as k-means clustering because considering only the last cluster in a prediction problem can be an efficient way to achieve accurate prediction. For example, Oh and Kim [8] obtained a good prediction performance using stock market data by referring to the last homogeneous clusters obtained from the data.

Meanwhile, the high variance problem using the last cluster can still present a problem during prediction because it is difficult to determine the size of the last cluster that ensures an accurate prediction. Hence, balancing the bias and the variance would be useful for the prediction problem using the last cluster. As mentioned above, adjusting the size of the last cluster would reduce the variance of a cluster obtained from partitioning data, which helps balancing the bias and the variance, so that the prediction accuracy would improve. Thus, in the current study, adjusting the size of the last cluster for the accurate prediction will be focused on.

Consider a function *f*(**x**) and its arbitrary estimator $\hat{f}(x)$. Then, the mean squared error (MSE) of $\hat{f}(x)$ is decomposed into $E[(f(x)-E[\hat{f}(x)]{)}^{2}]+E[(E[\hat{f}(x)]-\hat{f}(x){)}^{2}]$, which is a squared bias plus a variance. Hence, we can reduce the MSE by controlling the squared bias and the variance. However, unless *f*(**x**) is known, it is not easy to directly control the bias by specifying an estimator. Therefore, the method of reducing the variance can be more efficient. In addition, model regularization using a shrinkage method, such as least absolute shrinkage and selection operators (LASSO), is also popular for balancing bias and variance. In model estimation, these methods avoid the overfitting problem by adopting a new hyperparameter, termed a tuning parameter, that leads to increasing bias but decreasing variance through a smoothing effect. In general, this tuning parameter is usually obtained by cross-validation [9] and is helpful in alleviating the overfitting problem.

Aside from the model regularization methods, other unique approaches to the bias-variance balance in the econometrics community exist that address the trade-off between bias and variance with structural breaks in a dataset because structural breaks can be useful in determining where to partition a given dataset. Elliott [10] attempted to improve prediction accuracy by using weighted-averaging on the regression results for multiple structural breaks. Pesaran and Timmermann [11] presented some approaches based on structural breaks to determine a window (or cluster) size to achieve accurate prediction. Furthermore, also considering the structural breaks, Clark and McCracken [12] proposed a novel approach to improve the prediction accuracy by combining two estimated regression coefficients using a new hyperparameter, similar to LASSO.

The bootstrap method is well-known for reducing the bias [13] and variance [14] of an estimator. This method allows us to approximate an underlying functional form of a given dataset by averaging the noise of different bootstrapped samples out, thereby reducing bias. Furthermore, it is useful in obtaining an estimator with low variance through training a model multiple times. In particular, the relative advantage of variance reduction is often achieved at the expense of a slight increase in bias [15], which is one of the reasons why the bagging algorithm can improve model accuracy. Bauer and Kohavi [16] reported numerical results on the relatively greater variance reduction compared to bias reduction by using the bagging algorithm in a series of experiments. Fumera et al. [17] also showed that the bagging algorithm of *N* bootstrap replicates induces the same bias as that of a single bootstrap replicate but decreases the variance by a factor of *N*.

Therefore, combining the idea from a new model parametrization (such as the shrinkage method or the combination of two estimated regression coefficients) with the bootstrap method would be useful in balancing bias and variance, thus improving model prediction accuracy. Additionally, because a data-fitting-oriented model often leads to overfitting in many applications, it would be more efficient to adjust the size of the last cluster rather than to fit the entire dataset. In this study, the size-adjusted last cluster is termed as an adjusted last cluster (aCL), which has a size that falls between the size of the entire dataset and that of a last cluster by partitioning data. We expect this approach would improve the prediction performance of models based on the balance between bias and variance.

## Materials and methods

### Notations and assumptions

Here, we follow some conventional notations. Hat (^) notations such as $\hat{a}$ indicate an estimate, and an overbar (−) such as $\bar{a}$ denotes an average value.

Consider a given dataset $Z_{N}=\{Z_{n}:Z_{n}=(x_{n},y_{n})\in R^{m+1},n=1,\ldots ,N\}$, where $x_{n}={\left(x_{1n},x_{2n},\ldots ,x_{mn}\right)}^{T}\in R^{m},y_{n}\in R$, in which the *Z*_*n*_’s are arranged by the norm of **x**_*n*_, ‖**x**_*n*_‖, for prediction purposes. We want to determine the aCL to accurately predict *y*_*N*+*h*_ at **x**_*N*+*h*_ beyond **x**_*N*_, where *h* is the prediction horizon. To accomplish this, assume that **x**_*n*_ and *y*_*n*_ follow a data generating process (DGP) as follows:

$$y_{n}=f(x_{n})+e_{n},$$ (1)

where *e*_*n*_ is an independent and identically distributed random variable with zero mean, a variance of σ^2^ and is independent of **x**_*n*_.

Considering an estimator $\hat{f}(x_{n})$ based on **Z**_*N*_ for *f*(**x**_*n*_), our goal would then be to estimate $\hat{f}(x_{n})$ to accurately predict *y*_*N*+*h*_ at **x**_*N*+*h*_. The function $\hat{f}(x_{n})$ is usually obtained by minimizing the risk function as follows:

$$R(\hat{f}(x_{n}))=E\left[{\left(y_{n}-\hat{f}(x_{n})\right)}^{2}\right],$$ (2)

where E[∙] is the expected value.

Using $\hat{f}(x_{n})$, the prediction error (PE) is given by

$$PE(\hat{f}(x_{N+h}))=E\left[{\left(y_{N+h}-\hat{f}(x_{N+h})\right)}^{2}\right].$$ (3)

In the Results and Discussion section, in order to verify the method demonstrated in this paper, we will compare the prediction performances of the methods presented in this paper using the PE and test the statistical significance of the prediction performances.

### Bias and variance of a predictor: Whole dataset and subsample

In practice, the risk function is estimated as follows:

$$R(\hat{f}(x_{n}))=\frac{1}{N}\sum_{n=1}^{N}{\left(y_{n}-\hat{f}(x_{n})\right)}^{2}.$$ (4)

Considering a linear estimator $\hat{f}(x_{n})=\sum_{k=1}^{m}b_{k}x_{kn}=\beta^{T}x_{n}\;for\;\beta \in R^{m}$; the estimate is obtained by minimizing the risk function with respect to *β*:

$$\hat{\beta }=arg\;\min_{\beta \in R^{m}}\;R(\hat{f}(x_{n}))$$ (5)

$$={\left(X_{N}^{T}X_{N}\right)}^{-1}X_{N}^{T}y_{N},$$

where **X**_***N***_ = (**x**_1_,**x**_2_,…,**x**_*N*_)^*T*^ and ***y***_***N***_ = (*y*_1_,*y*_2_,…,*y*_*N*_)^*T*^. Then, the predictor is $\hat{f}(x_{N+h})={\hat{\beta }}^{T}x_{N+h}$.

**Fig 1:**
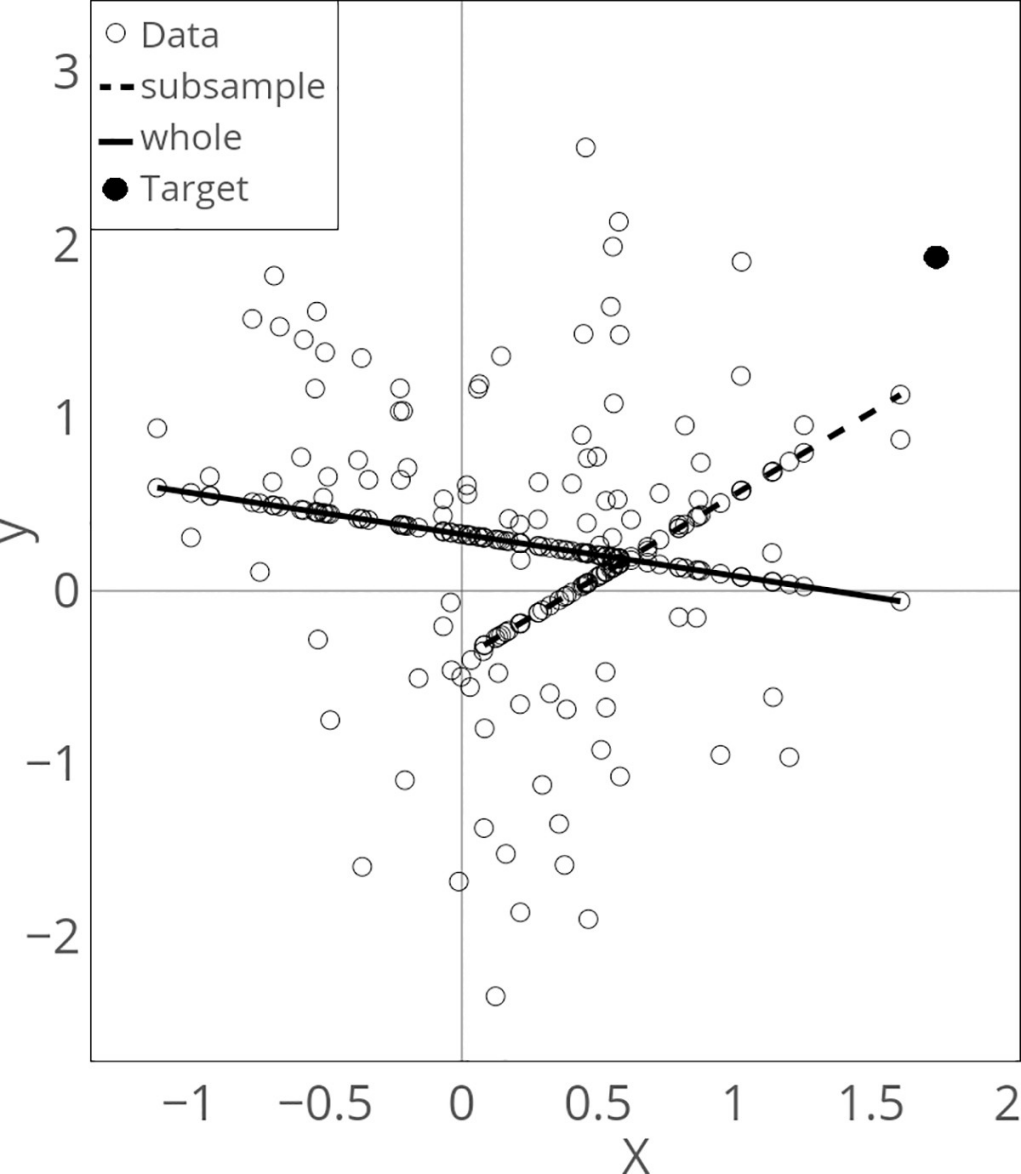
Prediction when using the whole dataset (whole, solid line) and a subsample (subsample, dashed line).

However, this predictor is based on the assumption that a linear estimator is applicable to the whole dataset; hence, this predictor produces a vulnerable prediction performance depending on the shape of the true function. For example, consider a DGP, *y*_*n*_ = −0.5*x*_1*n*_+*x*_1*n*_^2^+*e*_*n*_,*e*_*n*_~*N*(0,1) and a linear estimator $\hat{f}(x_{1n})=a_{0}+a_{1}x_{1n}$ over the whole dataset. Then, the value predicted by this estimator over the whole dataset would be quite distant from the prediction target as shown in Fig 1. However, the predicted value from a subsample of the given dataset achieves a more accurate prediction.

**Fig 2:**
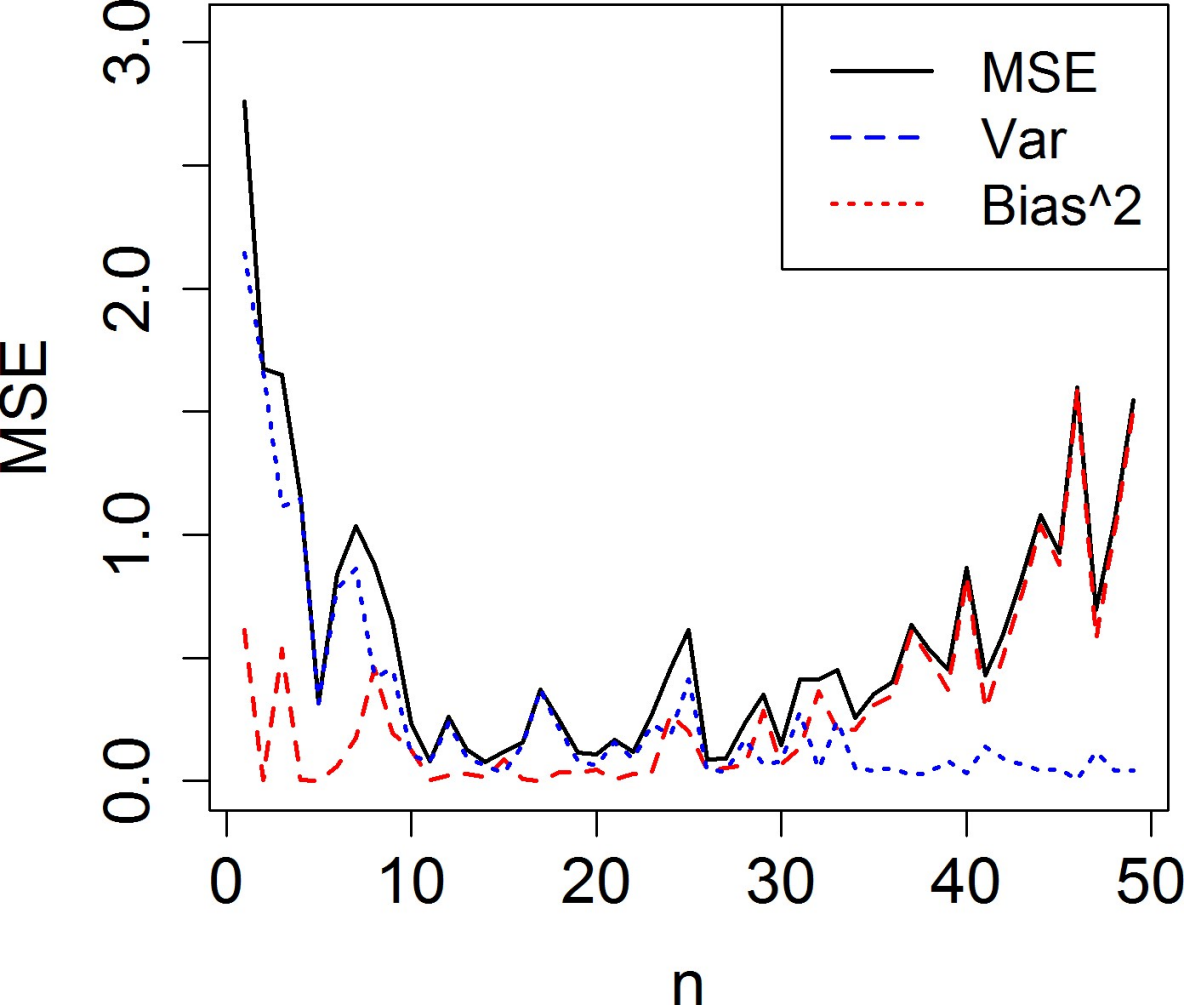
Change of MSE, squared bias (Bias^2) and variance (Var) of the predictor according to the number (n) of samples used in estimation. Here, the DGP and the estimator are the same as those in Fig 1.

Furthermore, as more samples are used, the bias of the predictor increases but its variance decreases, as shown in Fig 2. In general, an estimator from a subsample suffers from high variance, whereas the estimator from a whole dataset suffers from high bias [18]. Therefore, a size-adjusted subsample optimal for prediction should exist based on the balance between bias and variance [19]. For example, the size of such a subsample would be approximately 26, as seen in Fig 2.

To obtain the optimal subsample for prediction, first it is necessary to partition a given dataset using a method such as k-means clustering. However, determining the size of the last cluster for prediction may not be easy. To reliably determine the size of the last cluster, knowing the change points, structural breaks and local extrema of the given DGP can be useful. In practice, such values are not known *a priori*; one must guess the size of the last cluster by simply observing a graph of the DGP in an exogenous manner. However, partitioning the dataset in such a manner might be unreliable because the dataset observed is only a single realized sample among all the possible samples generated by the DGP. Thus, to obtain the size of the last cluster reliably, we would need to consider as many realized samples as possible, which would be unfeasible in practice. As an alternative, the bootstrap method used in [20] can be useful for virtually mimicking these samples to obtain the reliable last cluster.

### Adjusted last cluster for prediction

When simply partitioning one realized dataset, the size of the last cluster may be estimated to be smaller than the last cluster of the true function because the partitioning process of one realized dataset tends to be affected by noise, *e*_*n*_. To avoid this problem, adequately expanding the size of the last cluster results in reducing a predictor’s variance and would therefore be an alternative for approximating the last cluster of the true function. Hence, the aCL between the last cluster achieved by partitioning one realized dataset and the whole dataset would yield a more accurate prediction performance as long as the decreased variance by expanding the size of the last cluster exceeds the increased bias, similar to a regularization method such as LASSO [21].

**Fig 3:**
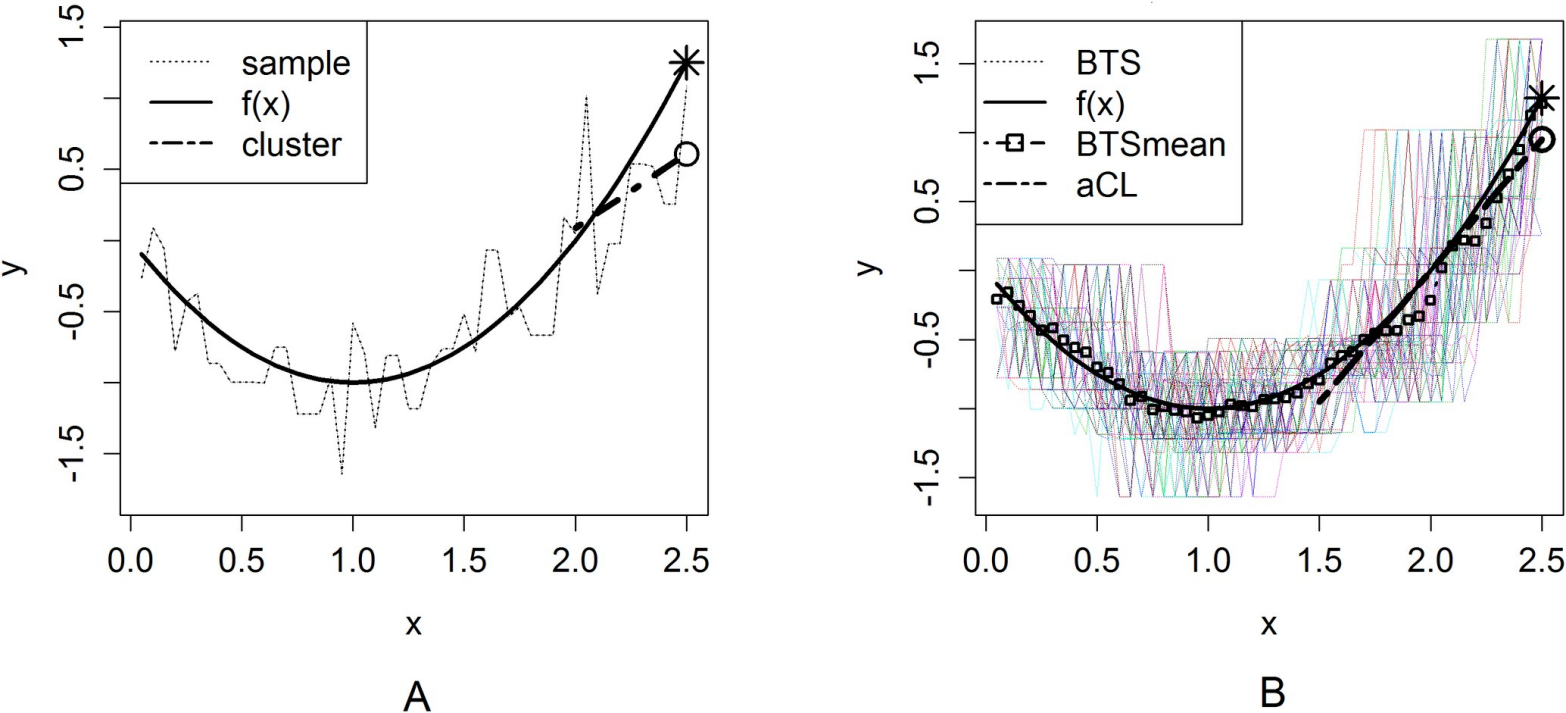
**Prediction based on the last cluster of one realized sample (A) and the last cluster of the new sample from bootstrapped samples (B).** The predictive value (circle) in 3B indicates the prediction target (asterisk) more accurately than does the predictive value (circle) in 3A. DGP: *y*_*n*_ = −2*x*_1*n*_+*x*_1*n*_^2^+*e*_*n*_,*e*_*n*_~*N*(0,1).

In the following example, Fig 3A depicts the prediction by one realized sample (dotted line). A true function (solid line) is *f*(x), and a linear estimator is used. The size of the last cluster (cluster, dashed line) from which a predictive value is obtained appears to be relatively small and is much influenced by noise, hence rendering a nonnegligible gap between the predictive value (circle) and the prediction target (asterisk). In contrast, Fig 3B shows that the new sample (BTSmean, dashed line with square) averaged over bootstrapped samples (BTS, dotted lines) well approximates the true function *f*(x). The last cluster (aCL, dashed line) based on the new sample is larger than the last cluster of the sample in Fig 3A. Thus, the predictive value of this last cluster more accurately indicates the prediction target.

Analogous to the idea of aCL, Clark and McCracken [12] proposed a novel approach to obtain a new regression coefficient for more accurate prediction by linearly combining two estimated regression coefficients under structural breaks: one estimated from a last rolling window (or a last cluster) and the other estimated from a recursive window (or a whole dataset).

Consider a sample **Z**_*N*_ realized from (1) and the linear estimator in (4)–(5), and denote the estimated regression coefficient obtained from the recursive window of size *N* as *β*_*R*_ and the estimated regression coefficient obtained from the last rolling window of size *L* as *β*_*L*_. Then, the linear combination of these two estimated regression coefficients *β*_*C*_ is

$$\beta_{C}=\alpha \beta_{L}+(1-\alpha )\beta_{R},\;0\leq \alpha \leq 1.$$ (6)

Hence, *β*_*C*_ is a compromise solution between *β*_*L*_ and *β*_*R*_. Clark and McCracken [12] showed that *β*_*C*_ allows more accurate prediction by inducing lower bias than *β*_*R*_ and lower variance than *β*_*L*_.

However, in [12], only one realized dataset was considered, and the regression coefficients already estimated from the last rolling window and the recursive window were used for *β*_*C*_ rather than being based on a last cluster optimal for prediction.

Therefore, if a sufficient number of realized datasets from a certain DGP are given and *β*_*C*_ is estimated from using these datasets, we can obtain a more generalized estimate for *β*_*C*_, which leads to more accurate prediction.

However, in practice, we cannot acquire many realized datasets from the DGP. Hence, in this paper, we adopt the bootstrap method to reproduce such datasets and achieve more accurate prediction as shown in Fig 3. We summarize the aCL method based on the bootstrap method as below.

**Algorithm** Prediction (one-step ahead) using the aCL

            1: Given a set $Z_{N}=\{Z_{n}:Z_{n}=(x_{n},y_{n})\in R^{m+1},n=1,\ldots ,N\}$

                      2: **for** *i*∈{1,…,*m*} **do**

        3: Reproduce the *i*-th bootstrap dataset, $Z_{N}^{i}=\{Z_{N}^{i}:Z_{N}^{i}=(x_{in},y_{in})\in R^{m+1},n=1,\ldots ,N\}$

4: Obtain a last cluster by partitioning the dataset (such as k-means clustering), and denote the size of the last cluster as *L*^*i*^

            5: Let the size of aCL be *C*^*i*^ = *α*_*i*_*L*^*i*^+(1−*α*_*i*_)*N*, 0≤*α*_*i*_≤1

6: Estimate ${\hat{C}}^{i}$ by minimizing the value of the risk function, ${\hat{C}}^{i}=arg\;\min_{L^{i}\leq C^{i}\leq N}\frac{1}{C^{i}}\sum_{n=N-C^{i}+1}^{N}{\left(y_{in}-\hat{f}(x_{in})\right)}^{2}$

                                7: **end for**

                          8: Calculate the average of the ${\hat{C}}^{i}’s$, $\bar{C}=\frac{1}{m}\sum_{i=1}^{m}{\hat{C}}^{i}$

        10: Estimate the regression coefficient using $\bar{C},\;{\hat{\beta }}_{C}=arg\;\min_{\beta_{C}\in R^{m}}\frac{1}{\bar{C}}\sum_{n=N-\bar{C}+1}^{N}{\left(y_{n}-\hat{f}(x_{n})\right)}^{2}$

                    11: Using ${\hat{\beta }}_{C}$, construct the predictor $\hat{f}(x_{N+1})$ for *y*_*N*+1_

Hence, the aCL acquired via the bootstrapped datasets is used for the predictor $\hat{f}(x_{N+1})$ rather than the last cluster obtained by simply partitioning the dataset, and it is expected to achieve a more accurate prediction performance based on the balance between bias and variance. The prediction performance of the presented method will be compared with other relevant methods in the Results and Discussion section.

### Methods for evaluating prediction error

In this section, we present several methods for comparing the prediction performance of the aCL method. First, we briefly introduce three existing optimization methods to check whether the aCL method improves the prediction performance of the optimization methods. Next, as competitors to the aCL method, we introduce three methods relevant to the aCL method. The numerical results of the prediction errors comparison are then presented in the Results and Discussion section.

#### Optimization methods

Because the aCL method is a way of searching for a last cluster optimal for prediction, we can apply it to general optimization methods. Therefore, we need to assess whether combining the aCL method with an optimization method reduces the prediction error of the optimization methods.

First, we combine the aCL method with ordinary least square (OLS), which is the most widely used optimization method due to its ease of use and easy interpretation. Under certain conditions, OLS is known to yield the estimator with the lowest variance among linear unbiased estimators [22]; however, in practice, it is not easy to create a linear unbiased estimator covering an entire dataset. However, a linear estimator can fit the data well when considering only a subsample of the entire dataset.

**Least squares adjusted with pseudo data.** The aCL method was next applied to a new optimization method for prediction—least squares adjusted with pseudo data (LSPD) [23]. The main principle of LSPD is to convert test error (or prediction error) into training error (or residual) by adopting a pseudo data point for a prediction target.

In general, to calculate the prediction target, the parameters of a predictive model are estimated by minimizing the training error using a given dataset, which can often lead to overfitting. Focusing only on a given dataset during optimization is one of the biggest reasons for the occurrence of the overfitting problem because it results in estimated parameters that fail to accurately predict the prediction target beyond the given dataset. Some methods using pseudo data beyond the given dataset have been suggested in the nonparametric community. For example, Schuster [24] suggested the reflection data method. When estimating kernel density with a dataset {*X*_1_,*X*_2_,…,*X*_*N*_}, this method claimed that adding a pseudo dataset {−*X*_1_,−*X*_2_,…,−*X*_*N*_} to the former could yield a consistent kernel density estimator based on the symmetry property of the kernel density function. Similarly, Cowling and Hall [25] proposed a general version of the reflection data method that can be used when discontinuities in the density derivatives exist.

However, unlike in kernel density function estimation, we are unable to use the symmetry property of a kernel density function in the prediction problem. There is little research on using pseudo data in the prediction problem, but Breiman [26] proposed a pseudo data method that reproduces the pseudo data from a certain convex combination of a given dataset to achieve more accurate prediction. Similarly, LSPD supposes that we can attain some pseudo data points close to the prediction target and add those to the given dataset to generate a new dataset. The estimated parameters based on the new dataset may then be useful for a more accurate prediction result by avoiding focusing only on the given dataset and thus alleviating the overfitting problem.

Put a pseudo data point $y_{N+1}^{p}$ close to *y*_*N*+1_ and denote the set including it by $Z_{N+1}^{p}=\{(x_{1},y_{1}),(x_{2},y_{2}),\ldots ,(x_{N},y_{N}),(x_{N+1},y_{N+1}^{p})\}$. Then, the following holds [23]:

$$E\left[{\left(y_{N+1}-\hat{f}(x_{N+1})\right)}^{2}|Z_{N+1}^{p}\right]\leq E\left[{\left(y_{N+1}-\hat{f}(x_{N+1})\right)}^{2}|Z_{N}\right],$$ (7)

where E[∙|**A**] is the conditional expected value given **A**.

In addition, among the various types of $y_{N+1}^{p}$, Kim et al. [23] reported that a sample mean of a given dataset usually results in the lowest prediction error. We use the sample mean for $y_{N+1}^{p}$ in this study.

**Least absolute shrinkage and selection operator.** Finally, we apply the aCL method to LASSO to investigate whether it still holds in the regularization method. LASSO generally provides a biased estimator due to penalization of the estimated coefficients [27]. Taking the previous example of a linear estimator used in (4)–(5), the LASSO method minimizes

$$\sum_{n=1}^{N}{\left(y_{n}-\sum_{k=1}^{m}b_{k}x_{kn}\right)}^{2}+\lambda \sum_{k=1}^{m}{|b}_{k}|,$$ (8)

where λ is the penalty (or tuning) parameter. 10-fold cross-validation is used to estimate λ in this study. The first term of (8) is the OLS method, which can possibly cause overfitting in a whole dataset, and the second term is the penalization term, which allows a predictive model to avoid the overfitting problem but leads to a biased estimator. Meanwhile, the aCL method uses a subsample of the given data; hence, it would not cause serious overfitting. In addition, based on this advantage, the penalization term would not shrink the estimated coefficients more than necessary, which alleviates the biased-estimator problem. Therefore, the aCL method is expected to reduce the prediction error when combined with LASSO.

#### Competing methods

**K-means clustering.** The k-means clustering algorithm is a well-known and widely used method of partitioning data. Hence, we evaluated whether the aCL method can reduce the prediction error compared to k-means clustering.

In general, k-means clustering is used for performing classifications; however, because it is based on unsupervised learning, k-means clustering is also useful for making predictions on real-world data problems [27, 28]. Meanwhile, the problem of selecting the appropriate k value in an application persists. Suppose that **Z**_*N*_ is partitioned into k subsets, S_1_,S_1_,…,S_*k*_. The k value is estimated as follows:

$$\hat{k}={\mathrm{argmin}}_{k\in Z^{+}}\sum_{i=1}^{k}\sum_{Z_{n}\in S_{i}}{∥Z_{n}-\mu_{i}∥}^{2},$$ (9)

where *μ*_*i*_ is the mean of S_*i*_.

Because the value of k is numerically determined in many cases [28], it is dependent on a type of data or on researcher knowledge. Moreover, the noise in a given dataset can easily lead to a small-sized cluster. Very small clusters result in high predictor variance, such that the prediction error increases beyond what is desirable [7]. Therefore, the aCL with a size between a last cluster and the whole dataset can result in lower prediction error than can k-means clustering.

**The new sample averaged over bootstrap samples.** As shown in Fig 3, the true function can be well approximated by bootstrap samples. Namely, a new sample averaged over the bootstrap samples can achieve a more accurate prediction performance than when simply using only one realized sample. Therefore, we need to evaluate the prediction performance based on the new sample averaged over the bootstrap samples compared to the aCL method.

**Linear combination of two coefficients.**As explained in the previous section, Clark and McCracken [12] suggested that the linear combination of two estimated regression coefficients improves the prediction performance, similar to the idea of the aCL method. However, in [12], only one realized sample was used, and a small number of change points were assumed. The aCL method is derived from using bootstrap samples and involves the last cluster among the multiple clusters in a given dataset; thus, the aCL method is expected to achieve a more accurate prediction result than the linear combination of two estimated regression coefficients.

## Results and discussion

### Simulation study

#### Simulation models

To examine whether the aCL method applies well to various functional forms, we conducted Monte Carlo simulations over four types of DGPs (100 Monte Carlo simulations for each DGP). The aCL method helps to find the change point of *f*(**x**), at which a whole sample is adequately partitioned so that a linear estimator fits well to the last cluster. Therefore, the DGPs having the change point would be good examples. DGP 1 is a univariate quadratic model, in which a change point exists at the local maximum. DGP 2 consists of two planes where the change points occur at the midpoint of a sample; thus the optimal location for partitioning data would be around the midpoint. DGP 3 is a hyperbolic paraboloid that resembles a saddle, in which a change point can be made from two directions. Thus, finding where to partition a given dataset is challenging. Finally, as a general example of DGP, we set DGP 4 to a multivariate polynomial function form. All the DGPs are estimated by a linear estimator as in (4)–(5):

$$\mathrm{DGP}\;1\;y_{n}=-3(\theta_{1}+\theta_{2})-2\frac{\theta_{1}}{\theta_{2}}x_{1n}+2\frac{\theta_{2}}{\theta_{1}}x_{1n}^{2}+e_{n}$$

$$\mathrm{DGP}\;2\;y_{n}=\left\{ \begin{array}{c} \frac{1}{\theta_{1}}-\frac{\theta_{1}}{\theta_{2}}x_{1n}+\frac{\theta_{1}}{\theta_{2}}x_{2n}+e_{n}\;for\;n\leq \frac{N}{2}\\ \frac{1}{\theta_{2}}-\frac{\theta_{2}}{\theta_{1}}x_{1n}+\frac{\theta_{2}}{\theta_{1}}x_{2n}+e_{n}\;for\;n>\frac{N}{2} \end{array}\right.$$

$$\mathrm{DGP}\;3\;y_{n}=\frac{1}{\theta_{1}}{x_{1n}}^{2}-\frac{1}{\theta_{2}}{x_{2n}}^{2}+e_{n}$$

$$\mathrm{DGP}\;4\;y_{n}=\frac{\theta_{1}}{\theta_{2}}x_{1n}+\frac{\theta_{2}}{\theta_{1}}x_{2n}+\theta_{1}x_{1n}x_{2n}+{\theta_{2}{x_{2n}}^{2}+e}_{n}.$$

To consider the dynamic structure in each DGP, we let *x*_*mn*_ follow the AR(1) structure for *m* = 1 and 2; *x*_1*n*_ = 0.5*x*_1*n*−1_+*e*_1*n*_,*e*_1*n*_~*N*(0,1) and *x*_2*n*_ = 0.7*x*_2*n*−1_+*e*_2*n*_,*e*_2*n*_~*N*(0,1), respectively. Moreover, to observe the variations in the numerical results according to the changes in coefficients, we generated the coefficients according to [29],

$$\theta_{k}=\sqrt{\frac{p}{1-p}\gamma_{k}},$$ (10)

$$\gamma_{k}=\left\{{\left(k/3\right)}^{A}{\left(3-k/3\right)}^{B}\right\}/\sum_{k=1}^{2}{\left\{{\left(k/3\right)}^{A}{\left(3-k/3\right)}^{B}\right\}}^{2},$$

where *p* takes the values {0.1,0.2,…,0.9}, and both *A* and *B* take the values {0.1,0.2,…,1}. The noise term *e*_*n*_ in each DGP follows an independent and identical normal distribution of zero mean and variances (denoted by Sig) of 1, 2, and 3. Additionally, we consider sample sizes (denoted by N) of 100, 200, and 300 and prediction horizons (denoted by Ho) of 1, 2, and 3. In brief, to test the prediction performance of the aCL method in a variety of situations, all DGPs were generated by Monte Carlo simulation with the independent variable of the AR(1) process, varying coefficients of DGP and variances of the noise term. In addition, we varied the sample size of DGP and prediction horizon to test the prediction performance of the proposed method.

Despite various simulation settings, these four DGP experiments may be insufficient to assert that the proposed method is always more accurate. For example, in the DGP consisting of only noise, a simple predictor of the sample mean would be a better choice; in contrast, in a DGP with no noise, 1-nearest-neighbor algorithm would achieve a good prediction result. However, for easily visible DGPs such as these extremal cases, one can directly specify an estimator for a given dataset.

As a statistical test on the prediction results among the presented methods we applied the Diebold-Mariano test [30], which is used to test the null hypothesis that two methods have the same prediction accuracy. This test is useful in many applications because it can be used for non-Gaussian or serially correlated data.

#### Results on the simulated data

**Fig 4:**
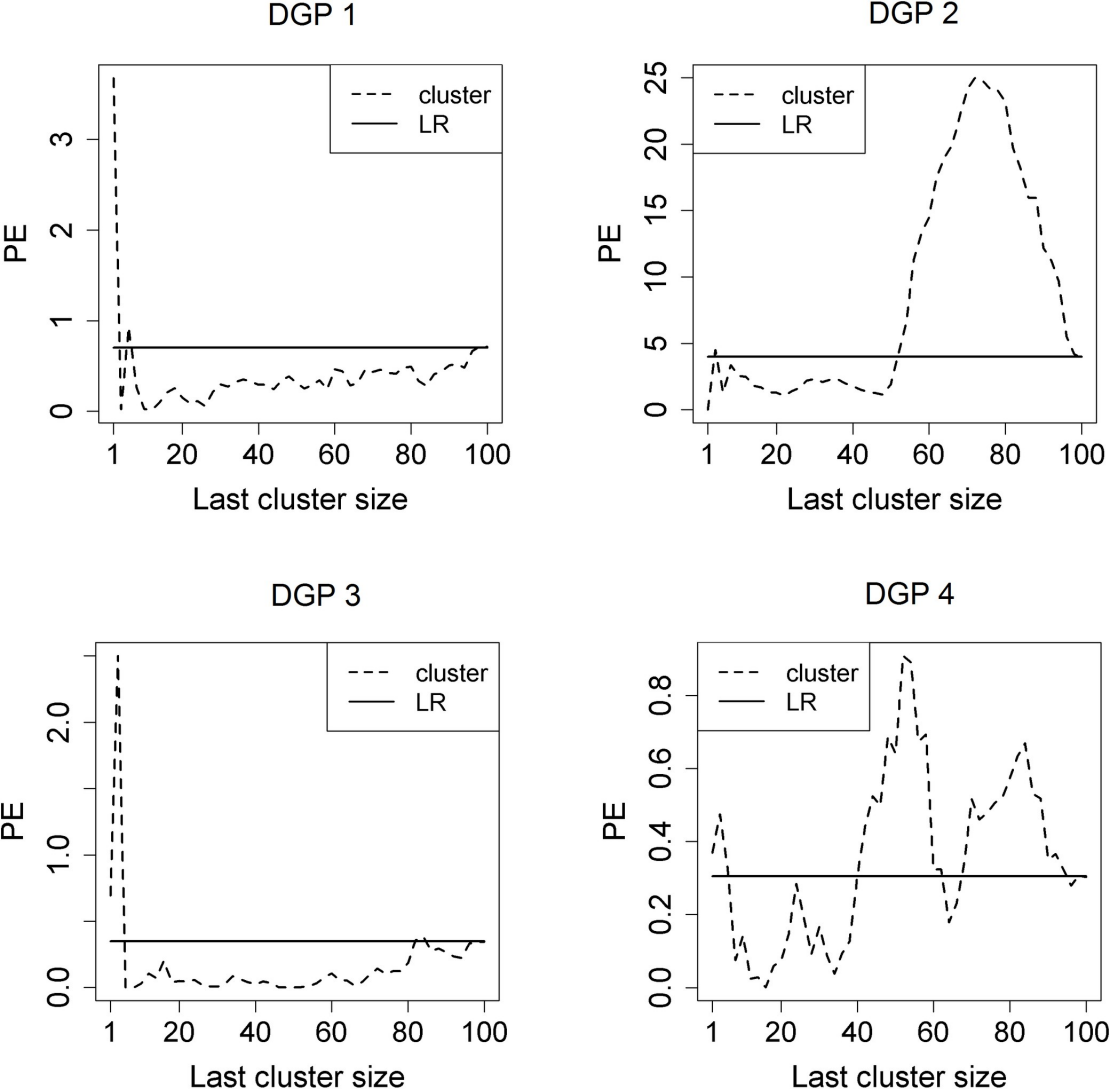
Change of the prediction error based on the size of the last cluster. Prediction based on the last cluster (cluster, dashed line) can yield lower prediction error than does linear regression over the entire dataset (LR, solid line). Because the full dataset is used for LR, the PE does not change in LR. For each DGP, **N = 100**.

First, it would be helpful to see the existence of the last cluster for more accurate prediction using each DGP. From Fig 4, we can observe that prediction based on some last clusters results in lower prediction error than does prediction using the entire dataset. Nevertheless, there exist other last clusters that yield higher prediction error than using the whole dataset. Therefore, selecting a last cluster of adequate size is important and so using the aCL method can be useful for obtaining the last cluster.

**Fig 5:**
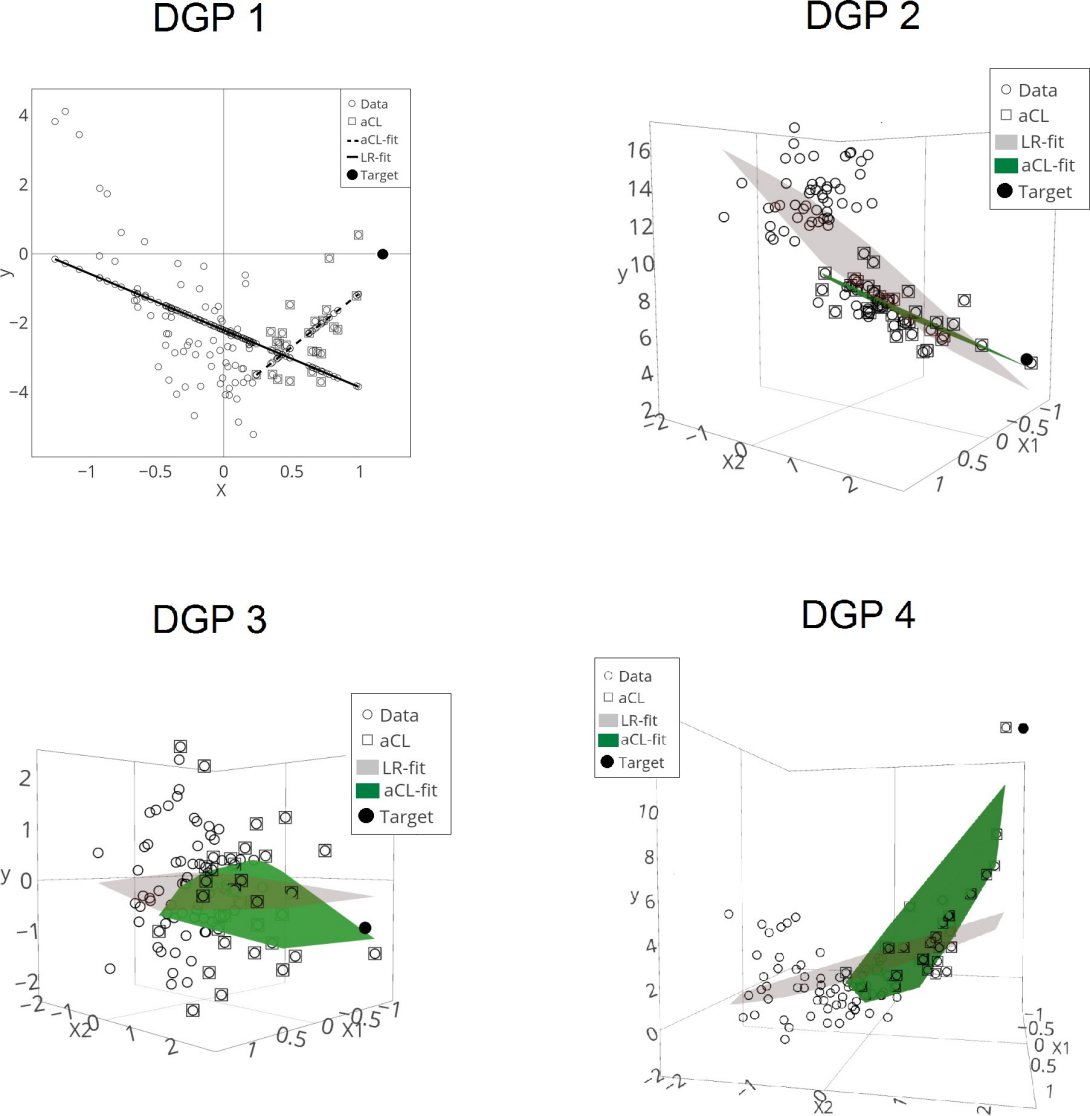
Selection of aCL and its prediction. DGP 1 shows how aCL (square) is selected among the data (white circle) and that the fitted line of aCL (aCL-fit, dashed line) more accurately indicates the prediction target (target, black circle) than does the linear regression fitted line of a whole dataset (LR-fit, solid line). DGP 2–4 also show that aCL is well selected in three-dimensional space, and the fitted plane of aCL (aCL-fit, dark green plane) more accurately indicates the prediction target than does the linear regression fitted plane of the whole dataset (LR-fit, light gray plane). For each DGP, N = 100, Ho = 1, and Sig = 1.

Furthermore, Fig 5 depicts how the aCL is chosen and the predictions are made based on the aCL. For all the DGPs, the estimator based on the aCL more accurately indicates the prediction target than does the estimator based on the entire dataset.

Tables 1–4 show the prediction errors of the methods used in this study. The first nine rows in the table present the results when the aCL method is applied to OLS, LSPD, and LASSO. The last six rows show the prediction errors of the competing methods (k-means clustering, the new sample obtained from bootstrap samples, and the linear combination of two estimated regression coefficients). Additionally, the tables present the changes of prediction errors according to the different sample sizes (N), horizons (Ho), and variances (Sig). The abbreviations in the tables are as follows. OLS = ordinary least squares, OLSaCL = OLS combined with aCL, LSPD = least squares adjusted with pseudo data, LSPDaCL = LSPD combined with aCL, LASSO = least absolute shrinkage and selection operator, LASaCL = LASSO combined with aCL, Kmeans = k-means clustering, Bootstr = the new sample obtained from bootstrap samples, and Comb = the linear combination of two estimated regression coefficients.

**Table 1:** Comparison of prediction error in DGP 1.

DGP = 1					N = 100									N = 200									N = 300				
Ho		1			2			3			1			2			3			1			2			3	
Sig	1	2	3	1	2	3	1	2	3	1	2	3	1	2	3	1	2	3	1	2	3	1	2	3	1	2	3
OLS	245.019	348.978	342.525	387.463	392.13	375.065	274.54	421.606	257.788	320.217	444.204	345.762	467.147	343.895	514.531	340.587	503.16	362.892	460.07	482.527	419.929	479.324	494.506	508.297	374.599	517.43	444.044
OLSaCL	11.608	14.307	17.168	20.125	20.842	27.083	20.693	31.435	27.26	13.484	26.915	25.485	37.781	19.109	63.072	23.303	44.693	29.793	27.662	32.758	30.162	52.051	44.51	63.703	28.637	45.64	38.795
(p-value)	(<0.001)	(<0.001)	(<0.001)	(<0.001)	(<0.001)	(<0.001)	(<0.001)	(<0.001)	(<0.001)	(<0.001)	(<0.001)	(<0.001)	(<0.001)	(<0.001)	(<0.001)	(<0.001)	(<0.001)	(<0.001)	(<0.001)	(<0.001)	(<0.001)	(<0.001)	(<0.001)	(<0.001)	(<0.001)	(<0.001)	(<0.001)
LSPD	246.171	346.157	340.285	381.899	385.778	370.38	271.444	412.312	255.431	322.563	446.452	347.975	468.145	345.49	515.003	342.05	502.971	364.028	461.974	484.368	420.827	480.497	495.739	509.549	374.776	517.988	444.158
LSPDaCL	20.039	27.033	28.1	32.241	30.671	36.312	30.084	42.633	34.552	16.839	41.457	30.424	58.335	21.882	80.442	26.411	60.528	33.179	42.287	47.008	37.065	70.264	61.544	88.701	35.353	65.583	44.794
(p-value)	(<0.001)	(<0.001)	(<0.001)	(<0.001)	(<0.001)	(<0.001)	(<0.001)	(<0.001)	(<0.001)	(<0.001)	(<0.001)	(<0.001)	(<0.001)	(<0.001)	(<0.001)	(<0.001)	(<0.001)	(<0.001)	(<0.001)	(<0.001)	(<0.001)	(<0.001)	(<0.001)	(<0.001)	(<0.001)	(<0.001)	(<0.001)
LASSO	243.205	341.971	339.174	384.461	389.413	372.398	272.355	418.626	253.913	318.275	441.989	344.245	464.411	342.197	511.615	338.708	500.569	361.098	457.355	479.373	407.125	476.788	490.855	488.41	372.313	515.099	441.486
LASaCL	12.787	19.905	18.788	20.27	21.737	28.358	21.306	33.839	26.819	14.698	29.041	27.258	42.372	19.072	71.371	22.328	39.689	27.366	26.81	32.928	32.569	53.885	46.549	59.386	28.678	47.937	38.953
(p-value)	(<0.001)	(<0.001)	(<0.001)	(<0.001)	(<0.001)	(<0.001)	(<0.001)	(<0.001)	(<0.001)	(<0.001)	(<0.001)	(<0.001)	(<0.001)	(<0.001)	(<0.001)	(<0.001)	(<0.001)	(<0.001)	(<0.001)	(<0.001)	(<0.001)	(<0.001)	(<0.001)	(<0.001)	(<0.001)	(<0.001)	(<0.001)
Kmeans	31.493	57.695	56.587	57.589	73.917	65.173	42.737	75.498	51.922	49.992	61.279	61.051	82.013	65.229	88.867	60.757	84.908	83.619	60.22	84.505	74.83	73.919	84.414	77.206	64.273	89.589	81.851
(p-value)	(0.002)	(<0.001)	(<0.001)	(0.001)	(<0.001)	(0.002)	(0.008)	(0.003)	(0.014)	(0.002)	(0.008)	(0.001)	(0.033)	(<0.001)	(0.084)	(0.008)	(0.002)	(0.002)	(0.005)	(0.004)	(<0.001)	(0.094)	(0.006)	(0.207)	(0.007)	(0.001)	(0.002)
Bootstr	242.343	346.982	341.665	388.084	390.902	374.487	276.42	421.122	259.228	321.503	445.978	348.669	470.8	347.044	517.961	340.979	506.693	363.502	463.408	484.174	422.316	480.895	496.311	509.644	375.228	520.118	444.081
(p-value)	(<0.001)	(<0.001)	(<0.001)	(<0.001)	(<0.001)	(<0.001)	(<0.001)	(<0.001)	(<0.001)	(<0.001)	(<0.001)	(<0.001)	(<0.001)	(<0.001)	(<0.001)	(<0.001)	(<0.001)	(<0.001)	(<0.001)	(<0.001)	(<0.001)	(<0.001)	(<0.001)	(<0.001)	(<0.001)	(<0.001)	(<0.001)
Comb	176.012	274.632	282.323	324.494	304.418	292.052	205.806	363.356	216.887	200.121	323.458	241.346	334.116	242.172	412.308	246.003	409.515	260.036	341.701	331.195	325.951	347.476	369.015	379.948	297.122	403.248	377.845
(p-value)	(<0.001)	(<0.001)	(<0.001)	(<0.001)	(<0.001)	(<0.001)	(<0.001)	(<0.001)	(<0.001)	(<0.001)	(<0.001)	(<0.001)	(<0.001)	(<0.001)	(<0.001)	(<0.001)	(<0.001)	(<0.001)	(<0.001)	(<0.001)	(<0.001)	(<0.001)	(<0.001)	(<0.001)	(<0.001)	(<0.001)	(<0.001)

NOTE: The three optimization methods (OLS, LSPD, and LASSO) are compared with their aCL methods. Other competing methods (Kmeans, Bootstr, and Comb) are compared with OLSaCL. The p-value of the Diebold-Mariano test is shown in parentheses.

**Table 2:** Comparison of prediction error in DGP 2.

DGP = 2					N = 100									N = 200									N = 300				
Ho		1			2			3			1			2			3			1			2			3	
Sig	1	2	3	1	2	3	1	2	3	1	2	3	1	2	3	1	2	3	1	2	3	1	2	3	1	2	3
OLS	1.312	1.262	3.423	2.445	2.055	5.018	1.567	2.245	6.831	4.172	7.628	11.998	4.761	8.751	21.282	5.275	10.754	16.625	6.184	10.507	20.276	6.852	14.483	21.081	6.955	12.511	28.861
OLSaCL	0.313	1.162	3.69	0.633	2.153	3.01	0.675	2.324	4.422	1.074	1.965	5.088	1.418	2.741	7.993	1.889	6.768	10.178	0.855	4.609	5.64	2.327	5.87	17.602	1.547	5.401	3.474
(p-value)	(<0.001)	(0.4)	(0.599)	(0.016)	(0.548)	(0.084)	(0.002)	(0.55)	(0.122)	(0.002)	(0.004)	(0.014)	(<0.001)	(<0.001)	(0.004)	(0.004)	(0.029)	(0.001)	(<0.001)	(0.01)	(<0.001)	(<0.001)	(<0.001)	(0.249)	(<0.001)	(0.016)	(<0.001)
LSPD	2.421	2.595	4.819	3.518	3.578	7.047	2.751	3.752	8.743	4.336	7.79	12.044	4.956	8.903	21.75	5.579	10.369	16.046	6.382	10.179	19.561	7.04	14.293	20.642	7.056	12.584	28.466
LSPDaCL	0.571	1.661	4.098	1.028	2.754	3.522	0.933	3.275	5.662	0.784	2.409	5.454	1.385	2.423	8.857	1.846	5.911	10.426	0.967	4.567	6.162	1.763	5.648	17.361	1.352	4.836	3.358
(p-value)	(<0.001)	(0.039)	(0.302)	(0.004)	(0.2)	(0.021)	(<0.001)	(0.262)	(0.114)	(<0.001)	(0.008)	(0.015)	(0.001)	(0.001)	(0.006)	(0.003)	(0.007)	(0.003)	(<0.001)	(0.011)	(<0.001)	(<0.001)	(<0.001)	(0.27)	(0.001)	(0.008)	(<0.001)
LASSO	1.287	1.175	3.27	2.073	2.277	5.035	1.477	1.958	6.175	3.96	6.821	10.83	4.518	8.244	20.203	5.162	10.429	15.718	6.116	10.57	19.652	6.753	13.408	20.884	6.907	11.668	28.137
LASaCL	0.424	1.354	4.409	0.803	2.369	2.036	0.531	3.048	3.2	0.989	1.714	4.673	1.526	2.721	8.551	1.922	7.845	13.449	0.854	4.364	5.92	2.097	6.621	17.856	1.408	5.57	3.317
(p-value)	(<0.001)	(0.406)	(0.563)	(0.034)	(0.356)	(0.018)	(0.001)	(0.462)	(0.08)	(0.001)	(0.003)	(0.016)	(<0.001)	(<0.001)	(0.006)	(0.005)	(0.043)	(0.001)	(<0.001)	(0.009)	(<0.001)	(<0.001)	(<0.001)	(0.175)	(<0.001)	(0.024)	(<0.001)
Kmeans	1.108	2.673	7.525	1.201	16.642	11.836	1.265	8.266	19.091	1.682	4.908	17.438	1.936	5.385	25.424	2.862	11.506	19.876	1.493	6.012	18.83	1.296	6.046	12.417	1.546	6.631	17.995
(p-value)	(0.001)	(0.051)	(0.014)	(0.105)	(0.078)	(<0.001)	(0.004)	(0.009)	(<0.001)	(0.096)	(0.012)	(0.017)	(0.171)	(0.047)	(0.002)	(0.157)	(0.116)	(0.039)	(0.005)	(0.208)	(<0.001)	(1)	(0.459)	(0.872)	(0.5)	(0.323)	(<0.001)
Bootstr	1.308	1.314	3.334	2.562	1.9	4.72	1.673	1.971	6.622	4.16	7.817	11.992	4.927	8.852	21.006	5.52	10.931	16.743	6.218	10.727	20.766	6.876	14.384	20.493	7.189	12.953	29.458
(p-value)	(<0.001)	(0.349)	(0.638)	(0.016)	(0.625)	(0.11)	(0.001)	(0.699)	(0.157)	(0.001)	(0.005)	(0.014)	(<0.001)	(0.001)	(0.005)	(0.003)	(0.024)	(0.001)	(<0.001)	(0.009)	(<0.001)	(<0.001)	(<0.001)	(0.286)	(<0.001)	(0.013)	(<0.001)
Comb	0.895	1.201	3.818	1.104	2.565	5.613	0.948	2.906	9.575	2.702	6.005	13.179	3.284	6.805	20.8	4.098	8.631	16.924	3.543	8.851	19.813	4.274	13.465	19.503	5.483	9.336	26.72
(p-value)	(0.002)	(0.461)	(0.457)	(0.128)	(0.335)	(0.023)	(0.272)	(0.164)	(0.013)	(0.009)	(0.005)	(0.022)	(0.019)	(0.003)	(0.006)	(0.017)	(0.177)	(0.015)	(<0.001)	(0.034)	(<0.001)	(0.019)	(0.001)	(0.346)	(0.002)	(0.081)	(<0.001)

**Table 3:** Comparison of prediction error in DGP 3.

DGP = 3					N = 100									N = 200									N = 300				
Ho		1			2			3			1			2			3			1			2			3	
Sig	1	2	3	1	2	3	1	2	3	1	2	3	1	2	3	1	2	3	1	2	3	1	2	3	1	2	3
OLS	11.451	14.406	20.64	12.451	17.555	24.14	11.901	17.448	22.579	11.881	11.586	19.696	12.124	16.382	16.467	11.457	14.385	27.168	13.35	13.871	17.449	15.314	14.568	15.742	17.187	16.616	19.512
OLSaCL	1.264	2.792	6.841	2.442	4.825	6.806	2.26	5.097	9.574	2.224	4.368	6.184	2.595	7.312	9.563	3.111	7.586	13.042	1.745	4.638	7.756	2.644	5.523	10.183	2.574	4.759	8.504
(p-value)	(<0.001)	(<0.001)	(<0.001)	(<0.001)	(<0.001)	(<0.001)	(<0.001)	(<0.001)	(<0.001)	(<0.001)	(<0.001)	(<0.001)	(<0.001)	(0.001)	(0.006)	(<0.001)	(0.004)	(0.003)	(<0.001)	(<0.001)	(<0.001)	(<0.001)	(<0.001)	(0.036)	(<0.001)	(<0.001)	(0.001)
LSPD	11.537	14.523	20.668	12.408	17.518	23.999	11.818	17.327	22.339	11.916	11.621	19.651	12.141	16.457	16.467	11.464	14.357	27.106	13.382	13.879	17.447	15.341	14.556	15.74	17.185	16.593	19.503
LSPDaCL	1.933	3.41	7.432	3.106	5.221	7.171	2.75	5.396	9.798	2.611	4.604	6.442	2.95	7.395	9.91	3.504	7.884	13.389	2.101	4.917	8.047	2.9	5.643	10.239	2.948	4.85	8.584
(p-value)	(<0.001)	(<0.001)	(<0.001)	(<0.001)	(<0.001)	(<0.001)	(<0.001)	(<0.001)	(<0.001)	(<0.001)	(<0.001)	(<0.001)	(<0.001)	(0.001)	(0.007)	(<0.001)	(0.005)	(0.003)	(<0.001)	(<0.001)	(<0.001)	(<0.001)	(<0.001)	(0.039)	(<0.001)	(<0.001)	(0.001)
LASSO	10.674	13.425	20.546	11.735	19.472	25.308	10.179	15.338	20.469	11.643	12.053	19.365	12.089	16.688	15.42	11.317	14.575	25.719	13.541	11.842	17.867	15.561	14.699	15.864	16.836	15.103	17.955
LASaCL	1.463	2.781	5.935	2.982	4.056	8.66	2.494	5.447	7.367	2.248	4.227	5.356	2.811	8.88	7.885	3.32	7.487	13.62	2.023	4.381	8.616	3.223	5.523	10.892	3.268	4.87	9.38
(p-value)	(<0.001)	(<0.001)	(<0.001)	(<0.001)	(<0.001)	(<0.001)	(<0.001)	(<0.001)	(<0.001)	(<0.001)	(<0.001)	(<0.001)	(<0.001)	(0.003)	(0.006)	(<0.001)	(<0.001)	(0.004)	(<0.001)	(<0.001)	(0.001)	(<0.001)	(<0.001)	(0.034)	(<0.001)	(<0.001)	(0.001)
Kmeans	2.563	7.893	10.698	3.343	8.103	16.749	4.24	20.004	18.19	4.522	8.317	18.469	4.543	15.137	14.435	4.616	9.554	26.893	3.158	7.748	11.102	4.257	5.532	9.018	5.339	8.582	18.172
(p-value)	(0.004)	(<0.001)	(0.018)	(0.019)	(0.006)	(<0.001)	(<0.001)	(0.002)	(0.024)	(0.001)	(0.011)	(<0.001)	(0.001)	(0.004)	(0.053)	(0.078)	(0.136)	(0.002)	(0.008)	(0.013)	(0.029)	(0.011)	(0.497)	(0.692)	(<0.001)	(0.03)	(<0.001)
Bootstr	11.041	14.159	20.696	12.14	16.87	22.999	11.782	17.205	22.1	11.971	11.623	19.506	12.064	16.029	16.159	11.421	14.514	27.127	13.919	14.261	18.269	15.648	14.829	15.88	17.714	17.178	19.675
(p-value)	(<0.001)	(<0.001)	(<0.001)	(<0.001)	(<0.001)	(<0.001)	(<0.001)	(<0.001)	(<0.001)	(<0.001)	(<0.001)	(<0.001)	(<0.001)	(0.001)	(0.006)	(<0.001)	(0.003)	(0.002)	(<0.001)	(<0.001)	(<0.001)	(<0.001)	(<0.001)	(0.036)	(<0.001)	(<0.001)	(<0.001)
Comb	10.029	12.552	15.673	10.116	11.488	18.54	10.705	16.353	20.803	10.821	11.194	18.972	11.045	15.652	15.623	10.55	13.2	26.992	10.366	12.985	16.602	12.654	13.66	14.458	14.695	15.628	18.763
(p-value)	(<0.001)	(<0.001)	(<0.001)	(<0.001)	(<0.001)	(<0.001)	(<0.001)	(<0.001)	(<0.001)	(<0.001)	(<0.001)	(<0.001)	(<0.001)	(0.002)	(0.012)	(<0.001)	(0.003)	(0.003)	(<0.001)	(<0.001)	(0.001)	(<0.001)	(<0.001)	(0.073)	(<0.001)	(<0.001)	(0.001)

**Table 4:** Comparison of prediction error in DGP 4.

DGP = 4					N = 100									N = 200									N = 300				
Ho		1			2			3			1			2			3			1			2			3	
Sig	1	2	3	1	2	3	1	2	3	1	2	3	1	2	3	1	2	3	1	2	3	1	2	3	1	2	3
OLS	7.727	7.565	7.016	8.059	7.505	6.873	9.74	12.159	8.514	17.703	21.741	27.373	17.958	25.376	29.965	18.701	24.961	31.936	25.405	34.045	47.398	25.205	35.533	52.104	23.552	35.046	44.619
OLSaCL	0.752	1.609	3.186	1.346	2.568	4.513	1.524	3.971	5.051	1.856	4.441	7.406	2.39	4.689	10.849	2.818	6.558	14.001	1.945	6.338	11.552	2.097	4.5	13.422	3.5	5.978	14.943
(p-value)	(<0.001)	(<0.001)	(0.001)	(<0.001)	(<0.001)	(0.033)	(<0.001)	(<0.001)	(0.053)	(<0.001)	(<0.001)	(<0.001)	(<0.001)	(<0.001)	(<0.001)	(<0.001)	(<0.001)	(<0.001)	(<0.001)	(<0.001)	(<0.001)	(<0.001)	(<0.001)	(<0.001)	(<0.001)	(<0.001)	(<0.001)
LSPD	7.645	7.669	7.386	8.23	7.559	7.11	9.793	12.046	8.912	17.89	21.194	27.522	17.928	25.051	30.201	18.731	24.976	32.115	25.697	33.569	47.688	25.52	35.904	51.636	23.865	34.523	45.203
LSPDaCL	1.492	2.258	3.612	2.436	2.929	4.545	2.744	4.795	5.728	2.473	5.033	7.928	2.912	5.367	11.446	3.164	7.062	14.802	2.673	6.921	12.695	2.764	4.703	14.555	4.197	6.684	15.923
(p-value)	(<0.001)	(<0.001)	(0.001)	(<0.001)	(<0.001)	(0.05)	(<0.001)	(0.001)	(0.086)	(<0.001)	(<0.001)	(<0.001)	(<0.001)	(<0.001)	(<0.001)	(<0.001)	(<0.001)	(<0.001)	(<0.001)	(<0.001)	(<0.001)	(<0.001)	(<0.001)	(<0.001)	(<0.001)	(<0.001)	(<0.001)
LASSO	7.798	8.409	7.049	8.028	7.566	7.109	9.745	12.414	6.997	17.788	21.601	27.361	18.011	25.394	30.045	18.797	24.946	33.342	25.353	34.16	47.734	25.223	35.593	52.959	23.629	35.68	45.011
LASaCL	0.888	1.239	2.863	1.387	2.459	5.283	1.716	3.931	5.214	1.88	4.459	6.735	2.416	5.624	12.326	3.382	6.999	14.528	1.909	5.764	11.203	2.321	4.76	13.978	3.581	6.929	15.242
(p-value)	(<0.001)	(<0.001)	(0.001)	(<0.001)	(<0.001)	(0.058)	(<0.001)	(<0.001)	(0.085)	(<0.001)	(<0.001)	(<0.001)	(<0.001)	(<0.001)	(<0.001)	(<0.001)	(<0.001)	(<0.001)	(<0.001)	(<0.001)	(<0.001)	(<0.001)	(<0.001)	(<0.001)	(<0.001)	(<0.001)	(<0.001)
Kmeans	1.822	4.847	13.545	2.355	8.571	16.997	2.598	6.659	18.116	4.42	7.092	21.05	4.729	8.856	25.98	6.308	14.677	26.239	4.737	10.346	20.862	4.838	11.242	22.267	5.026	10.693	23.504
(p-value)	(0.001)	(<0.001)	(<0.001)	(0.009)	(<0.001)	(<0.001)	(0.008)	(0.006)	(<0.001)	(<0.001)	(0.002)	(<0.001)	(0.006)	(0.002)	(<0.001)	(<0.001)	(<0.001)	(0.002)	(<0.001)	(0.036)	(0.004)	(0.001)	(<0.001)	(0.005)	(0.072)	(0.001)	(0.01)
Bootstr	7.654	7.491	7.192	8.057	7.487	7.006	9.63	11.909	8.531	17.907	21.882	27.061	18.274	25.895	29.923	18.877	25.004	32.182	26.497	35.249	49.32	26.247	37.054	53.803	24.284	36.392	46.379
(p-value)	(<0.001)	(<0.001)	(0.001)	(<0.001)	(<0.001)	(0.025)	(<0.001)	(<0.001)	(0.05)	(<0.001)	(<0.001)	(<0.001)	(<0.001)	(<0.001)	(<0.001)	(<0.001)	(<0.001)	(<0.001)	(<0.001)	(<0.001)	(<0.001)	(<0.001)	(<0.001)	(<0.001)	(<0.001)	(<0.001)	(<0.001)
Comb	5.273	5.866	6.986	6.253	6.683	8.126	7.583	7.632	8.257	15.247	16.04	24.833	15.289	19.234	28.538	15.627	20.16	29.114	18.257	31.635	40.538	18.814	28.54	50.077	16.133	32.559	39.39
(p-value)	(<0.001)	(<0.001)	(0.001)	(<0.001)	(<0.001)	(0.009)	(<0.001)	(0.001)	(0.037)	(<0.001)	(<0.001)	(<0.001)	(<0.001)	(<0.001)	(<0.001)	(<0.001)	(<0.001)	(<0.001)	(<0.001)	(<0.001)	(<0.001)	(<0.001)	(<0.001)	(<0.001)	(<0.001)	(<0.001)	(<0.001)

The results in Tables 1–4 indicate that the three aCL methods outperform their preceding optimization methods under different conditions. Considering the type of DGP, the prediction errors are relatively lower in the curved functional forms (DGP 1, 3, and 4) compared to DGP 2, which comprises two planes. Additionally, when Sig is large in some cases, the aCL method shows relatively low prediction performance. Regarding the prediction errors of the three competing methods, we also observe that the aCL method mainly shows lower prediction errors than those of the three competitors. It is quite evident that the aCL method yields lower prediction errors than do Bootstr and Comb. Meanwhile, k-means clustering yields good prediction results when N = 300 in DGP 2, where the effect of noise is relatively low, and so k-means clustering can work well [31].

In addition, because the size of the aCL is between the whole dataset and a last cluster, the variance of prediction error when using the aCL method is lower than that when using k-means clustering, as presented in Table 5, and so the aCL method can help to obtain a stable prediction performance.

**Table 5:** Variance of prediction errors by the aCL method and k-means clustering.

	DGP 1	DGP 2	DGP 3	DGP 4
OLSaCL	13.884	3.680	3.047	5.964
Kmeans	14.875	7.365	6.344	7.438

NOTE: Prediction errors from Tables 1–4 are used

**Fig 6:**
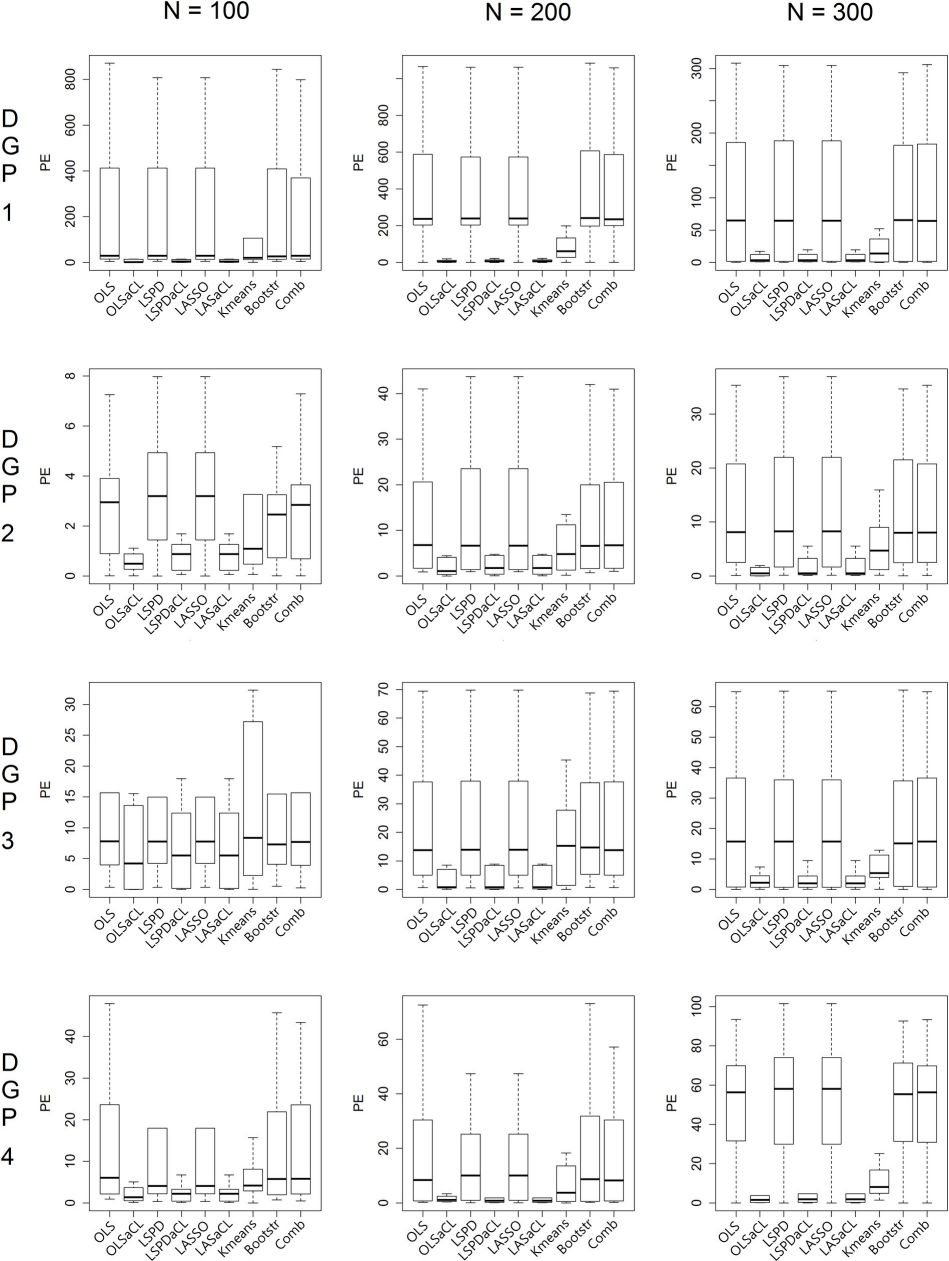
Prediction error comparison by sample size (Ho = 2,Sig = 2).

To demonstrate the robustness of the aCL method under various conditions, boxplots of the prediction errors are presented according to N, Ho, Sig, and coefficients. First, changes of the sample size N have little influence on the performance of the aCL method compared to its preceding optimization method and competing methods, as seen in Fig 6. As N increases, the aCL method consistently shows lower prediction errors than the other methods. K-means clustering achieves the second-lowest prediction errors in many cases but sometimes causes very high prediction errors.

**Fig 7:**
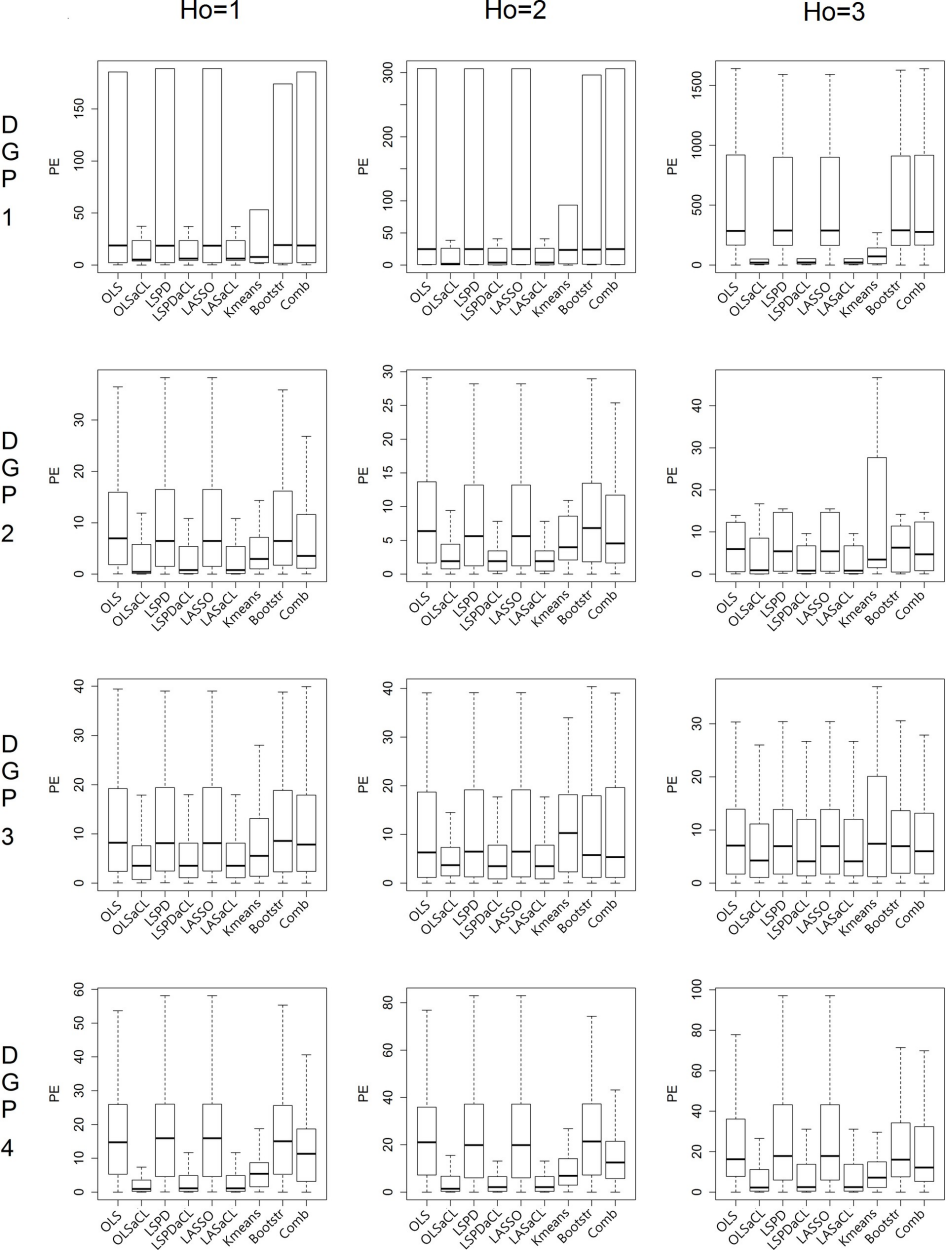
Prediction error comparison by horizon (N = 200,Sig = 2).

As in the previous example, the aCL method outperforms the other methods for all the prediction horizons, as shown in Fig 7. A longer prediction horizon should affect the prediction ability of a prediction model more than a shorter prediction horizon, and so we can observe that prediction errors increase as Ho increases. In particular, k-means clustering shows high prediction errors when Ho is high.

**Fig 8:**
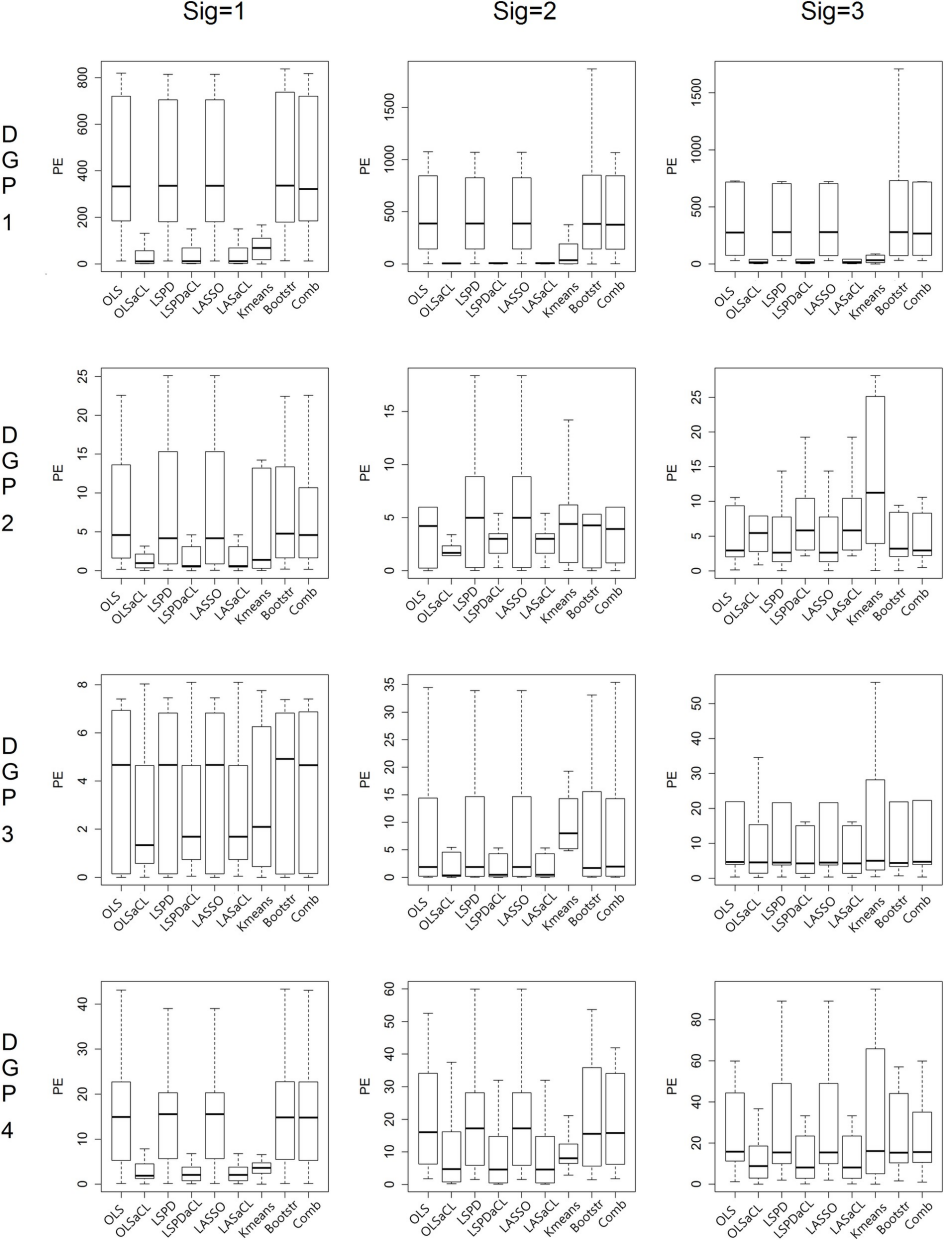
Prediction error comparison by variance (N = 200,Ho = 2).

A high variance of noise generally degrades prediction ability. Because the size of a cluster is smaller than the size of the full dataset, methods using the cluster would be considerably influenced by the size of the variance. In Fig 8, we can see that the prediction performance of the aCL method decreases as the variance increases. In particular, k-means clustering produces very poor prediction performances in cases with increasing variance.

**Fig 9:**
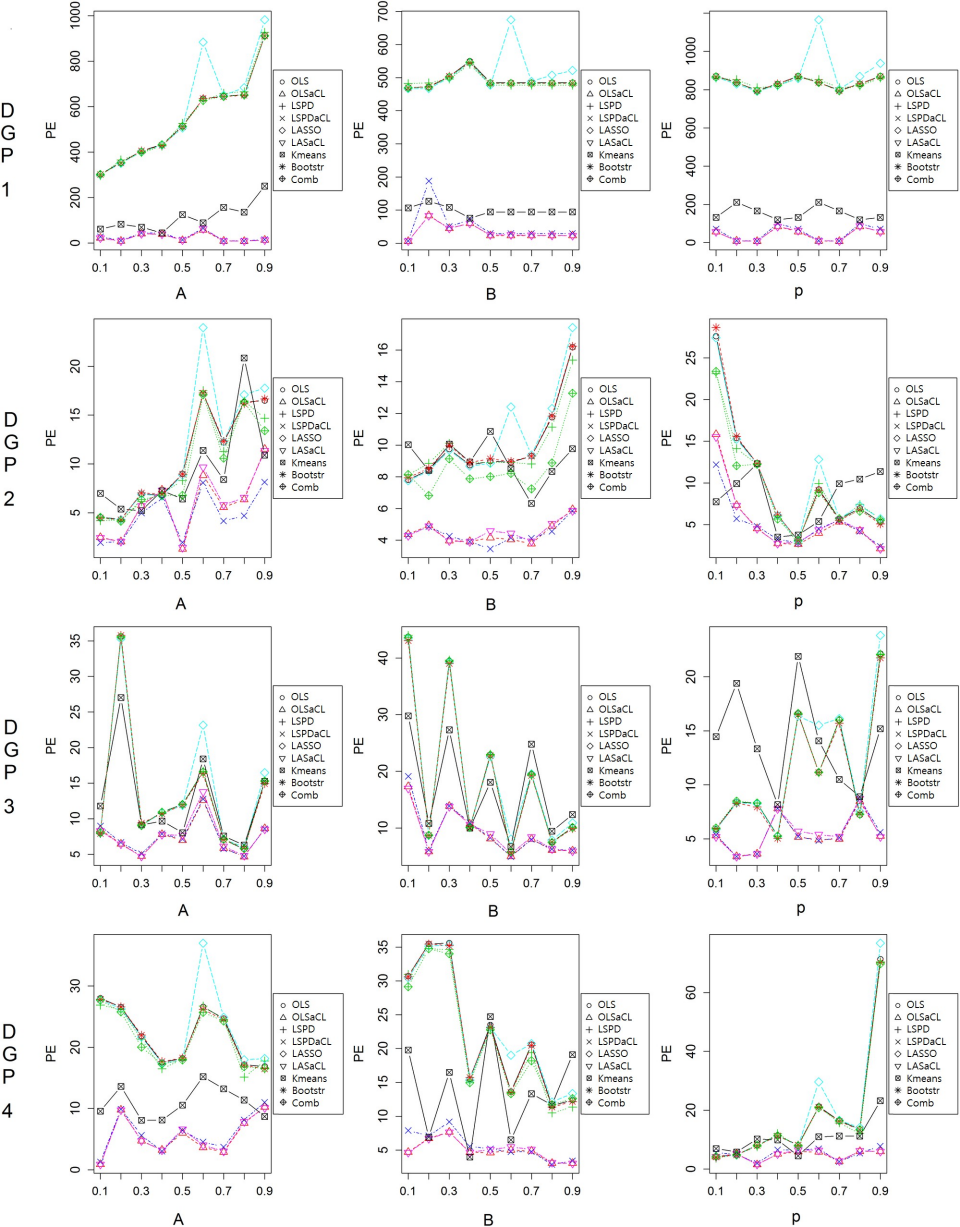
Prediction error comparison by changes of coefficients.

In Fig 9, we present the prediction errors resulting from changes of coefficients. Similar to previous experiments, the aCL methods achieve lower prediction errors than do the other methods. The prediction errors of k-means clustering also seem low in some cases; however, its prediction performance appears to fluctuate depending on the change of coefficients.

**Estimation of change point.** This section discusses whether a change point of a DGP is well approximated by the aCL method when compared to k-means clustering. Because a change point is distinctly set as half the sample size in DGP 2, we use the data generated by DGP 2. For the robustness check, the change point is estimated across the various values of N, Ho, and Sig. Well-estimated change points should be approximately half of the sample size. As shown in Table 6, the change points estimated by the aCL method are close to the true change points, whereas the change points estimated by k-means clustering are downward-biased because k-means clustering usually produces a small cluster size by the effect of the noise.

**Table 6:** Change points estimated by OLSaCL and Kmeans (DGP 2).

Factor	Value	OLSaCL	Kmeans
	100	44.5	18.8
N (Ho = Sig = 1)	200	100.5	25.2
	300	147.2	48.7
	1	41.3	18.2
Ho (N = 100,Sig = 1)	2	47 1	6.1
	3	50.4	1.5
	1	43 1	6.4
Sig (N = 100,Ho = 1)	2	40.5	24.0
	3	45.0	18.7

NOTE: The change point is the average over Monte Carlo iterations

**Fig 10:**
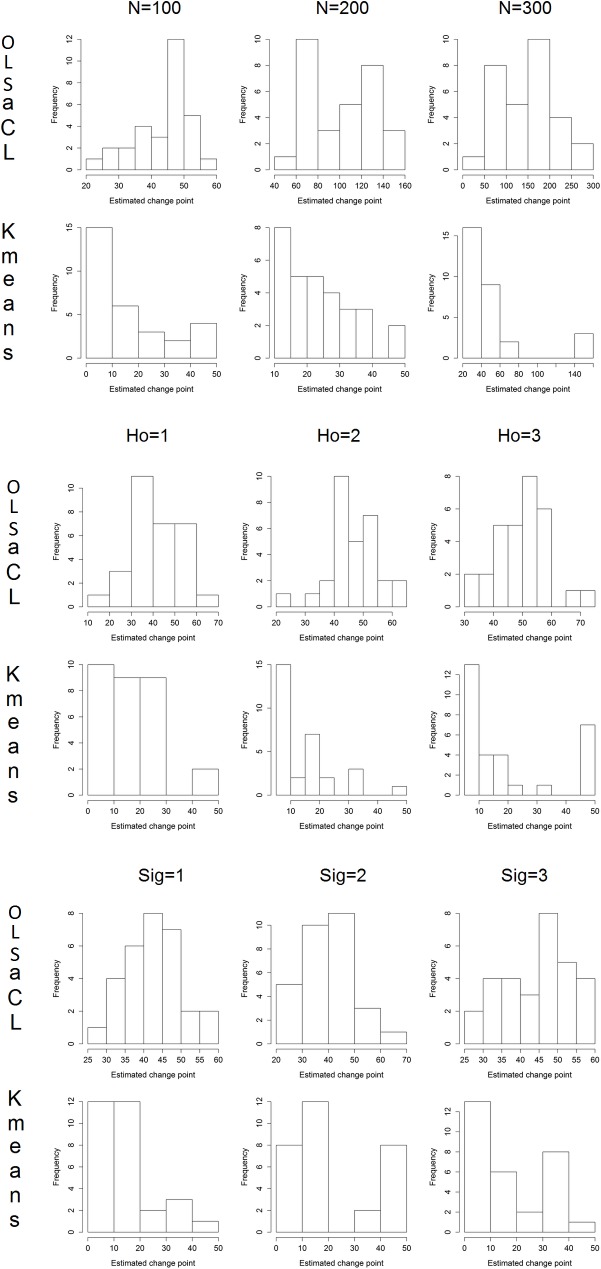
Histograms of change points estimated by OLSaCL and Kmeans.

Fig 10 depicts the histograms of estimated change points by OLSaCL and Kmeans through 100 Monte Carlo iterations. We can see that the histogram of OLSaCL shows a central tendency similar to a normal distribution, unlike Kmeans.

### Real data study

#### Stock market index

As the first application using real data, the S&P 500 index is predicted by the USD/EUR exchange rate and interest rate (effective federal funds rate in the US). Financial data have been the subject of numerous studies. In particular, the relation between stock market index and the exchange rate has long been studied [32, 33]. Additionally, the interest rate is known to affect the stock market because it is usually considered as a substitute for stock market returns [34].

More recently, machine learning methods such as the nearest neighbor algorithm have been applied to predict the stock market index [35], the exchange rate [36], and the interest rate [37] by focusing on the nonlinear characteristics of financial data. Meanwhile, most financial data have stylized facts that make prediction difficult [38], such as the fat tail distribution, clustering volatility, etc. These financial data characteristics frequently result in unpredictable spikes or clusters in time-series data which degrade the prediction power of a model using the whole dataset. Therefore, the nearest neighbor algorithm tends to be much influenced by the outliers leading to difficulty in establishing the predictive model, which might bring about the varying prediction performances depending on the range of the dataset [37]. Therefore, partitioning a dataset can be effective for these types of data. Some works reported improved prediction performances by adopting the k-means clustering method [39, 40, 41]. However, most of the related works estimate the number of clusters numerically and directly use the obtained clusters. So one may be able to test the many numbers of clusters until the desirable result is obtained [41], which makes the size of the cluster somewhat unreliable. On the other hand, because the aCL method applies the bootstrap method, it is expected to yield more reliable results.

We adopted a dataset comprising a total of 250 weeks of data from Sep. 30, 2011 to Sep. 23, 2016. To control the effect of the data size, the dataset is divided into six equal-sized subsets. Each subset includes 100 instances, from which the last 30 are predicted. The first-order difference data are used as in the usual financial data analysis [42].

**Fig 11:**
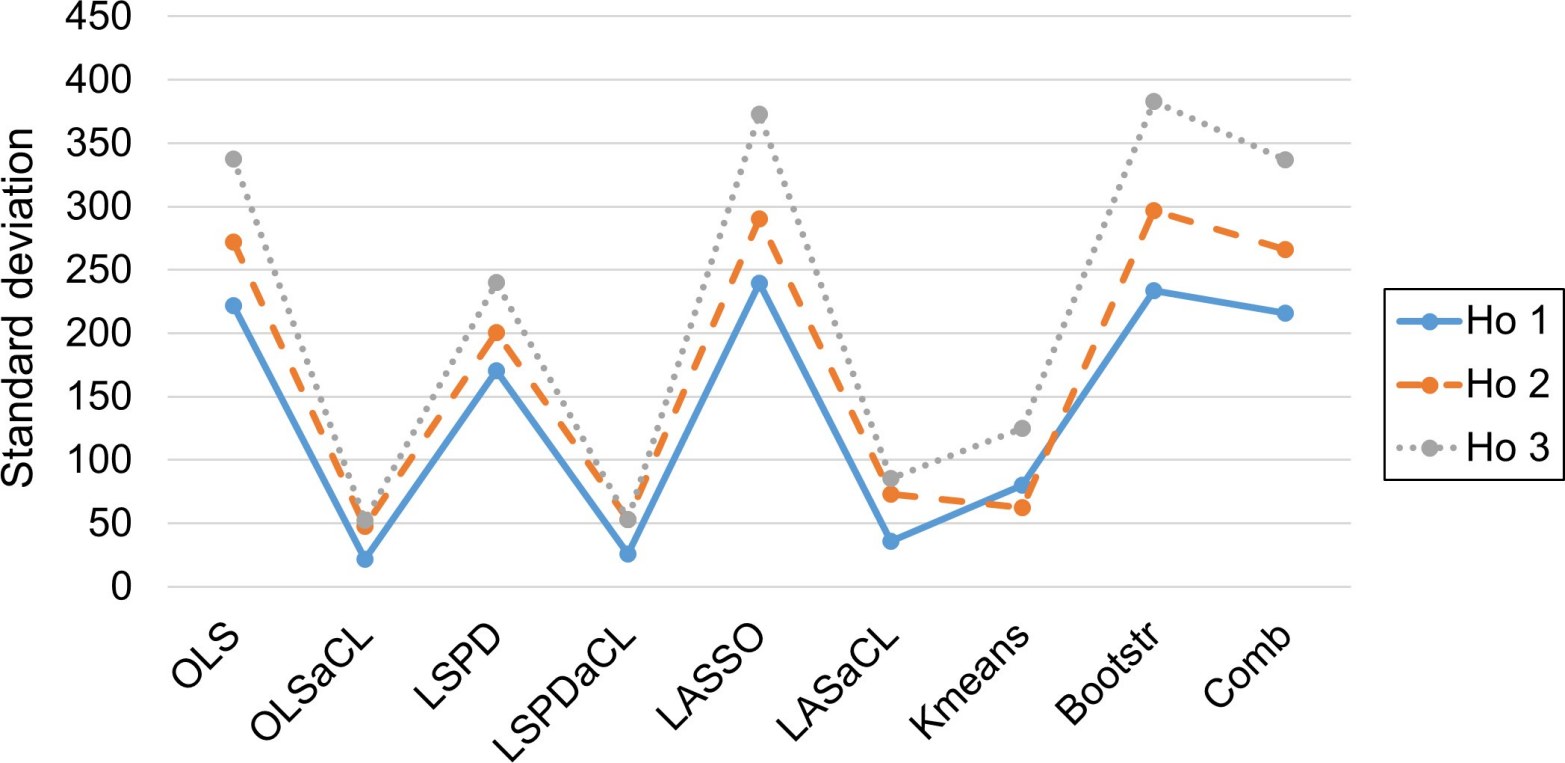
Comparison of standard deviations of prediction errors over the prediction horizons.

Given a dataset of size *N*, we predict the values at *N*+1,*N*+2 and *N*+3. As seen in Table 7, the aCL methods largely outperform their preceding optimizations and competing methods. K-means clustering yields very poor prediction performances in several cases, for example, in the third subset; similarly, the prediction performance of the aCL method is relatively low in the third subset compared to others. Note that standard deviations in prediction errors of the aCL methods over all the prediction horizons are lower than other methods (Fig 11), which implies the low variance of the prediction error in the aCL methods.

**Table 7:** Comparison of prediction error (S&P 500 index).

Subset	1			2			3			4			5			6		
Ho	1	2	3	1	2	3	1	2	3	1	2	3	1	2	3	1	2	3
OLS	1,623.641	1,456.661	1,583.565	1,006.735	1,044.876	795.876	5,150.994	5,351.771	5,600.641	4,570.235	4,343.653	4,392.129	5,056.139	4,986.251	4,904.958	5,006.36	4,930.956	4,933.17
OLSaCL	529.096	424.337	243.216	553.64	333.66	357.637	610.101	196.099	266.285	2,213.057	1,671.478	1,543.156	950.783	895.095	548.783	658.434	827.888	1,213.569
(p-value)	(<0.001)	(<0.001)	(<0.001)	(<0.001)	(<0.001)	(<0.001)	(<0.001)	(<0.001)	(<0.001)	(<0.001)	(<0.001)	(<0.001)	(<0.001)	(<0.001)	(<0.001)	(<0.001)	(<0.001)	(<0.001)
LSPD	1,542.055	1,392.232	1,494.012	1,110.979	1,149.076	935.288	4,816.755	4,994.519	5,216.836	3,933.531	3,770.43	3,783.216	4,315.94	4,268.341	4,188.265	4,197.11	4,142.642	4,132.846
LSPDaCL	522.096	448.278	292.519	633.418	419.701	459.249	296.197	85.188	125.423	1,542.501	1,196.477	1,049.984	664.488	644.935	338.042	450.153	599.778	866.493
(p-value)	(<0.001)	(<0.001)	(<0.001)	(<0.001)	(<0.001)	(<0.001)	(<0.001)	(<0.001)	(<0.001)	(<0.001)	(<0.001)	(<0.001)	(<0.001)	(<0.001)	(<0.001)	(<0.001)	(<0.001)	(<0.001)
LASSO	1,389.534	1,257.328	1,354.365	777.932	803.751	596.907	3,980.313	4,116.747	4,200.481	4,428.887	4,716.819	4,279.51	4,713.972	4,792.391	4,648.729	6,022.143	5,743.751	5,356.878
LASaCL	640.095	324.586	191.067	479.821	291.952	273.487	528.755	171.586	203.63	1,982.531	3,512.937	1,270.665	2,552.292	912.82	690.8	2,249.648	2,826.237	1,796.834
(p-value)	(<0.001)	(<0.001)	(<0.001)	(<0.001)	(<0.001)	(<0.001)	(<0.001)	(<0.001)	(<0.001)	(<0.001)	(0.167)	(<0.001)	(<0.001)	(<0.001)	(<0.001)	(<0.001)	(<0.001)	(0.099)
Kmeans	2,106.306	1,528.799	1,642.423	896.124	1,432.912	196.805	687.919	935.397	2,028.618	8,666.065	8,603.418	7,978.947	5,706.817	5,039.112	5,268.619	4,690.76	4,681.7	4,832.931
(p-value)	(<0.001)	(<0.001)	(<0.001)	(<0.001)	(<0.001)	(1)	(0.327)	(0.03)	(0.004)	(<0.001)	(<0.001)	(<0.001)	(<0.001)	(<0.001)	(<0.001)	(<0.001)	(<0.001)	(<0.001)
Bootstr	1,627.618	1,459.733	1,534.486	964.687	959.824	711.141	5,115.023	5,308.184	5,607.416	5,142.519	4,557.184	4,470.688	5,229.428	5,405.18	5,419.757	5,263.469	4,759.555	5,259.559
(p-value)	(<0.001)	(<0.001)	(<0.001)	(<0.001)	(<0.001)	(<0.001)	(<0.001)	(<0.001)	(<0.001)	(<0.001)	(<0.001)	(<0.001)	(<0.001)	(<0.001)	(<0.001)	(<0.001)	(<0.001)	(<0.001)
Comb	1,626.753	1,457.796	1,584.437	1,380.856	1,475.762	1,351.905	4,892.685	5,209.421	5,214.018	1,915.456	1,845.557	1,836.637	2,128.457	2,083.357	2,070.776	1,731.392	1,700.678	1,706.512
(p-value)	(<0.001)	(<0.001)	(<0.001)	(<0.001)	(<0.001)	(<0.001)	(<0.001)	(<0.001)	(<0.001)	(0.91)	(0.01)	(<0.001)	(<0.001)	(<0.001)	(<0.001)	(<0.001)	(0.001)	(0.148)

#### Home price index

The home price index (S&P/Case-Shiller U.S. National Home Price Index) is predicted by using the mortgage rate (30-year fixed rate mortgage average in the US) and the expected inflation (survey by University of Michigan).

A total of 250 monthly data points from Feb. 1990 to Dec. 2015 are included, and the data are divided into the six equal subsets as in the previous example. The last 30 instances out of 100 instances in each subset are predicted, and the prediction horizon ranges from 1 to 3.

There exists a vast literature predicting home prices. Many studies on this topic adopt time-series analysis tools [43, 44]. With regard to the factors influencing home price, the expected inflation can have a positive effect on home prices because the inflation rate usually reflects home prices [45]. However, a rise in inflation results in an increase in interest rates and a corresponding reduction in home prices [46]. Hence, the expected inflation has different influences on home prices depending on the relevant economic environment. In general, the mortgage rate is known to have a negative influence on home prices [47, 48]. As the time-series model enables achieving a good prediction performance, the selection of the lag parameter in the time-series model still remains a factor that is not easy to be determined. Moreover, as previous studies [43, 44] adopted the method of combining the different models in order to obtain the stable prediction performance, there also still remains the problem about how to combine models or which models to choose. Therefore, a clustering algorithm could be an alternative for the prediction problem when it is not easy to determine a comprehensive time-series model explaining the whole dataset.

**Fig 12:**
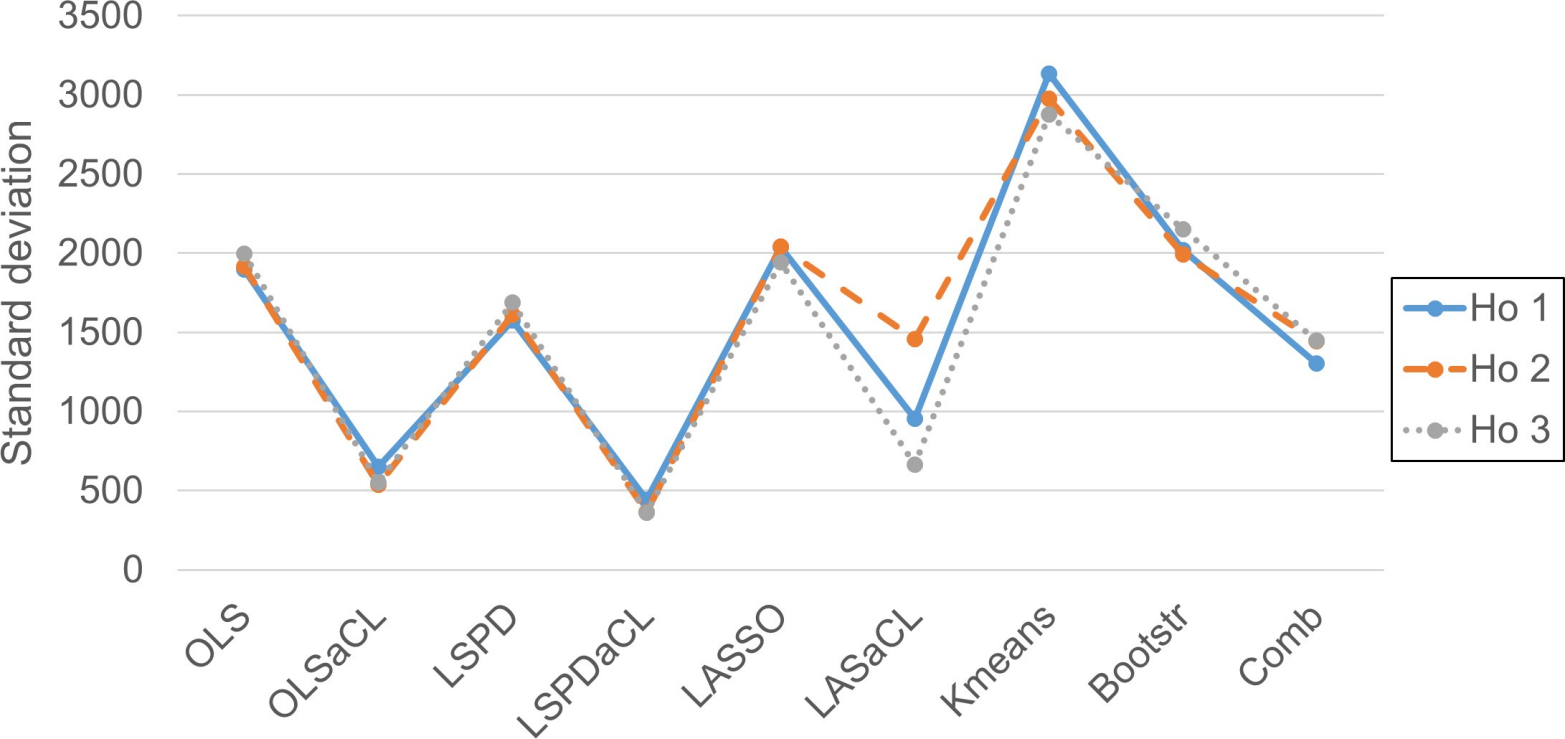
Comparison of standard deviations of prediction errors over the prediction horizons.

Similar to the case of the stock market index, we can also check the low standard deviations of prediction errors of the aCL methods in Fig 12.

**Fig 13:**
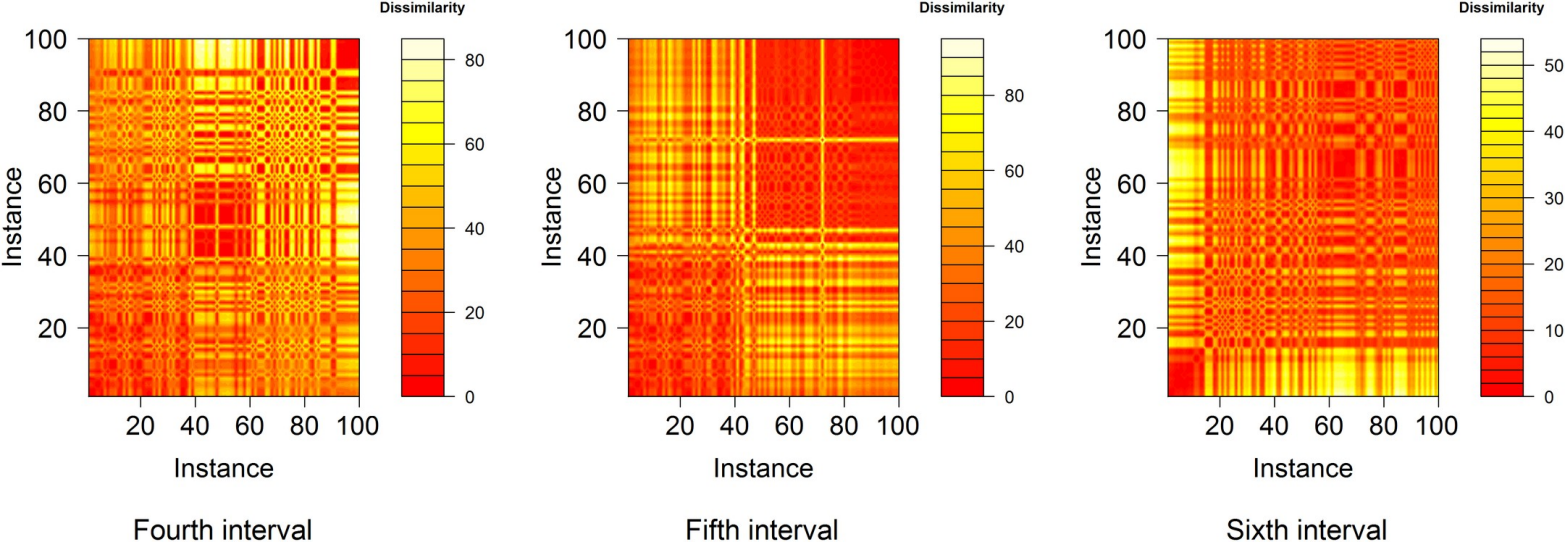
Heat maps of the dissimilarity matrix of instances from the fourth to the sixth subset. The dissimilarity metric is the Euclidean distance between the instances.

Table 8 shows that the aCL methods mainly achieve good prediction performances; however, in the fourth and sixth subsets, the aCL method is unable to yield good prediction performances and the k-means clustering also results in high prediction errors. For this phenomenon, a clue can be found in the heat maps in Fig 13, which show the dissimilarity matrix of the instances from the fourth to the sixth subsets. In the fifth subset, where the aCL method results in low prediction error, the instances from approximately the 50th to 100th appear well-clustered according to the dissimilarity level; thus, the prediction targets from the 70th to the 100th instances can be predicted well by the aCL methods. In contrast, in the fourth and sixth subsets, the instances around the prediction targets do not show well-clustered shapes; thus, we can conclude that methods using the clustering algorithm do not work well at these subsets.

**Table 8:** Comparison of prediction error (Home price index).

Subset	1			2			3			4			5			6		
Ho	1	2	3	1	2	3	1	2	3	1	2	3	1	2	3	1	2	3
OLS	7.828	10.981	13.477	104.179	122.637	159.995	535.21	675.131	860.787	27.34	22.612	27.207	405.931	433.311	477.964	72.431	59.753	68.189
OLSaCL	1.415	3.605	13.186	14.503	59.207	88.479	16.915	89.869	156.927	22.389	37.38	59.258	35.495	36.581	27.162	63.628	139.413	99.749
(p-value)	(0.008)	(0.125)	(0.489)	(<0.001)	(<0.001)	(<0.001)	(<0.001)	(<0.001)	(<0.001)	(0.258)	(0.934)	(0.999)	(<0.001)	(<0.001)	(<0.001)	(0.216)	(0.964)	(0.724)
LSPD	12.156	16.989	19.974	77.154	88.39	113.302	402.313	491.3	607.367	9.664	14.131	17.752	330.297	348.942	380.806	75.899	63.338	71.756
LSPDaCL	3.056	2.571	0.64	20.338	52.732	65.159	26.75	85.028	125.856	3.74	4.397	10.576	26.787	26.469	18.86	74.36	139.601	106.155
(p-value)	(0.001)	(0.009)	(0.027)	(<0.001)	(<0.001)	(<0.001)	(<0.001)	(<0.001)	(<0.001)	(0.014)	(0.006)	(0.134)	(<0.001)	(<0.001)	(<0.001)	(0.442)	(0.971)	(0.762)
LASSO	6.664	9.908	12.219	106.23	123.888	159.673	585.304	728.003	961.346	23.275	20.403	24.667	413.923	437.733	477.003	79.21	64.433	76.155
LASaCL	1.274	2.98	9.705	33.559	78.001	87.183	24.095	129.964	233.149	20.15	30.896	43.616	82.133	48.193	26.764	90.636	201.612	148.198
(p-value)	(0.011)	(0.066)	(0.398)	(<0.001)	(<0.001)	(<0.001)	(<0.001)	(<0.001)	(<0.001)	(0.258)	(0.934)	(0.999)	(<0.001)	(<0.001)	(<0.001)	(0.832)	(1)	(0.907)
Kmeans	17.135	28.166	65.534	25.309	38.026	89.703	52.252	120.376	302.769	97.486	121.879	184.943	4.441	4.316	1.604	218.579	157.704	296.049
(p-value)	(<0.001)	(<0.001)	(<0.001)	(<0.001)	(1)	(0.432)	(0.025)	(0.235)	(0.001)	(0.005)	(0.01)	(0.053)	(1)	(1)	(1)	(<0.001)	(0.329)	(0.001)
Bootstr	9.311	14.371	13.942	118.511	136.676	171.779	582.779	758.247	993.253	29.41	24.612	29.126	396.858	421.812	466.379	72.878	61.841	67.481
(p-value)	(0.006)	(0.093)	(0.473)	(<0.001)	(<0.001)	(<0.001)	(<0.001)	(<0.001)	(<0.001)	(0.191)	(0.896)	(0.998)	(<0.001)	(<0.001)	(<0.001)	(0.203)	(0.96)	(0.733)
Comb	7.795	10.854	13.35	99.291	117.107	155.867	516.993	657.46	858.033	27.171	22.242	27.019	403.307	429.392	475.835	74.532	60.194	68.216
(p-value)	(0.008)	(0.127)	(0.494)	(<0.001)	(<0.001)	(<0.001)	(<0.001)	(<0.001)	(<0.001)	(0.26)	(0.942)	(0.999)	(<0.001)	(<0.001)	(<0.001)	(0.165)	(0.965)	(0.726)

## Conclusions

Partitioning data produces a more accurate prediction than using a whole dataset when a true function is not known or when setting an estimator for the whole dataset is difficult. Meanwhile, the small-sized cluster produced by simply partitioning data causes high variance problems, which leads to high prediction errors. In this study, we showed that adjusting the size of the last cluster avoids high prediction error. To adjust the size of the last cluster between the whole dataset and the last cluster, we applied the bootstrap method, which has the effect that a given model is trained multiple times by different bootstrapped samples generated from a certain DGP; hence, the model can estimate the reliable location of the last cluster that is optimal for prediction. Therefore, we believe that this paper contributes to understanding that the adjusted last cluster could be optimal for prediction based on the balance between bias and variance by the bootstrap method.

In this paper, the aCL method was shown to reduce prediction errors using the numerical results of both simulated and real data. Notice that the aCL method is not for establishing a complete model fitting a whole dataset but for selecting an optimal subsample for prediction. Therefore, an estimator of simple functional form such as a linear function can be easily used with the aCL method for prediction, and this advantage of the aCL method would serve a practical use in research or application. In addition, the aCL method yielded the size-adjusted last cluster by the bootstrap method, which is easy to be adopted in studies using a clustering algorithm, and thus produces a more reliable subsample for prediction.

Nevertheless, this study has some limitations. The datasets were numerically partitioned, which usually leads to the problem of time consumption that occurs with other clustering algorithms. As described in the second real data example, for the subsamples not suitable for the clustering algorithm, the k-means clustering yielded very high prediction errors; hence, the aCL method was also unable to achieve a good prediction performance. To solve this problem, a more elaborate technique needs to be developed. However, observing the degree of clustering in data before using a clustering algorithm could be helpful to avoid this problem. In addition, as future work, a theoretical study on the aCL method should be done to rigorously prove the reliability of the proposed method.

In summary, we proposed a new method that adjusts the size of a last cluster to achieve accurate prediction. By applying the bootstrap method to find the most reliable aCL, this method could yield low prediction errors. More specifically, the aCL method can be useful in many applications using clustering algorithms for a stable prediction performance.
